# A systematic review of wild grass exploitation in relation to emerging cereal cultivation throughout the Epipalaeolithic and aceramic Neolithic of the Fertile Crescent

**DOI:** 10.1371/journal.pone.0189811

**Published:** 2018-01-02

**Authors:** Alexander Weide, Simone Riehl, Mohsen Zeidi, Nicholas J. Conard

**Affiliations:** 1 Institute for Archaeological Sciences, University of Tübingen, Tübingen, Germany; 2 Tübingen Senckenberg Center for Human Evolution and Palaeoenvironment, University of Tübingen, Tübingen, Germany; 3 Department of Early Prehistory and Quaternary Ecology, University of Tübingen, Tübingen, Germany; Missouri Botanical Garden, UNITED STATES

## Abstract

The present study investigates the occurrence of wild grasses at Epipalaeolithic and aceramic Neolithic sites in the Near East in order to assess their role in subsistence economies alongside the emergence of cereal cultivation. We use Chogha Golan in the foothills of the central Zagros Mountains (ca. 11.7–9.6 ka cal. BP) as a case study, where the archaeobotanical data suggest the frequent exploitation of a complex of wild grasses for almost 2,000 years. Domesticated emmer replaced these wild grasses as the major food resources towards the end of occupation at the site (ca. 9.8 ka cal. BP). We discuss possible implications of this development and conclude that the traditional concept of pre-domestication cultivation seems unsuited for explaining the patterns from Chogha Golan. These data are in good accordance with the overall picture in the Zagros Mountains, where wild grasses were routinely gathered throughout the early Holocene. In contrast, wild grasses were gradually replaced by wild cereals in the Levantine corridor since the end of the Pleistocene. However, several sites located in this region provide evidence for a continuous exploitation of wild grasses alongside emerging cereal cultivation and most of these taxa were part of the earliest segetal floras that evolved with the appearance of domestic cereals throughout the 11^th^ millennium cal. BP. Some sites contemporary to the Pre-Pottery Neolithic B still provide evidence for the usage of wild grasses, which possibly reflects the utilization of edible arable weeds and continuous gathering of wild grasses by more mobile groups.

## Introduction

The aceramic Neolithic in the Near East is characterized by the emergence of sedentary farming communities and marks a major change in human subsistence economies. Although still disputed [1–4], many scholars regard the “Neolithic Revolution” as a mosaic-like and protracted process, which is characterized by distinct trajectories towards farming in different sub-regions of the Fertile Crescent [5–19]. Archaeobotanical research on the emergence of agriculture has long focused on the origins and identification of domesticated cereals [20–24], which represents a major research area until today [25–27]. The last 20 years saw major advances in methodological and theoretical approaches, which eventually resulted in the proposition of the pre-domestication cultivation hypothesis [28–32]. This scenario assumes that the focus on wild cereals at sites in the southern and northern Levant during the early Pre-Pottery Neolithic (PPN) reflects the beginnings of cultivation, although morphological signs of domestication are still rare or absent. Besides evidence for increasing grain sizes over time [33–35] and the gradual replacement of gathered wild species by wild progenitors [32,36], this hypothesis is based on the interpretation of potential arable weeds as indicators for cultivation activities [37,38].

Today we owe this intensive archaeobotanical research a much clearer picture of emerging cultivation than during the 1990s and substantial methodological advances for the analysis of Neolithic plant assemblages. However, such a heavy focus on emerging cereal cultivation and domestication automatically tends to neglect the exploitation of wild plant resources by early farming communities. Many authors acknowledged the presence of a high diversity of potentially usable wild plants in aceramic Neolithic assemblages, but predominantly interpreted fruits and nuts from *Pistacia*, *Ficus*, *Vitis*, *Capparis* or *Amygdalus* as gathered foods [23,28,32,35,39–41]. This is mainly due to the fact that these fruit-bearing trees, shrubs and climbers produce large, calorie-rich fruits and do not contribute to crop-processing products or by-products. In contrast, many potentially usable wild plants are part of arable weed floras and their fruiting structures often accumulate in archaeobotanical assemblages consisting of threshing and sieving remains. Disentangling gathered species and arable weeds therefore represents a major challenge in interpreting prehistoric archaeobotanical assemblages [42–44].

Based on finds from Ohalo II, Hayonim cave and Kebara cave we know that wild plant foods, including grasses and legumes, have been exploited by Middle Palaeolithic and Epipalaeolithic hunter-gatherers [45–47]. Particularly Ohalo II yielded a rich archaeobotanical assemblage with a high proportion of wild grasses. Weiss et al. interpreted these finds as representative of staple foods that have been gathered alongside wild cereals and formed part of the “broad spectrum revolution” originally postulated by Flannery in 1969 [48–50]. Additional evidence for the frequent exploitation of wild Poaceae species that have never been domesticated came from sites in the eastern wing of the Fertile Crescent. Savard et al. found high proportions of wild grains in the early Holocene assemblages from Qermez Dere and M’lefaat [51], supporting the view of Weiss and his colleagues that non-cereal taxa played an important role in subsistence economies prior to the beginnings of cultivation. Similarly surprising were the storage finds of wild grains from PPNA Gilgal and the aceramic levels at Çatalhöyük, suggesting that wild grasses have been continuously consumed alongside cultivated wild and possibly even domestic cereals [52,53]. However, Weiss et al. demonstrated a decrease in abundance for non-cereal taxa towards the PPNA in the Levant, having negligible proportions during the PPN and being insignificant for the diet of the earliest farmers [48]. This assumption was in accordance with the overall development of early Holocene subsistence strategies in the Levantine corridor, but at the same time raises the question of whether this view is applicable to the aceramic Neolithic as a whole? Particularly as scholars seem to agree upon a substantial diversity in subsistence strategies that characterize the late Pleistocene and early Holocene human groups in the Near East [12,18,51,54–60]. Moreover, several rich archaeobotanical assemblages from the Levantine corridor have been investigated and published since 2004, making it sensible to re-evaluate the relation between wild grass use and emerging cereal cultivation in this region.

### Focus and goals of the study

In this paper we present additional evidence for the routine exploitation of wild grasses alongside wild cereals from the aceramic Neolithic site of Chogha Golan in the foothills of the central Zagros Mountains. Based on this case study we discuss how the emergence of agriculture on the site relates to the gathering of wild grains that directly “competed” with domesticated cereals in the subsistence economy. In this regard, our main goals are to investigate how the roles of wild grasses and wild and domestic cereals change throughout the site’s occupation and whether the traditional pre-domestication cultivation hypothesis is suited to explain this development.

By using Chogha Golan as a starting point, we systematically reviewed the occurrence of wild grasses at Epipalaeolithic and aceramic Neolithic sites in the entire Fertile Crescent. Our main goal is to trace the development of wild grass exploitation in relation to emerging cereal cultivation and domestication. For investigating this, we conducted a spatial and temporal analysis on the occurrence of wild grasses and cereals. In addition to proportions and ubiquity values, we considered the interpretation of the wild grasses by the individual authors and their occurrence in specific contexts. The major aims of these analyses are to evaluate how the role of wild grasses changed through time and whether we see differences in plant exploitation strategies among the sub-regions of the Fertile Crescent. This is of major significance for understanding the Neolithic transition, because the harvests of wild grasses directly compete with cultivated grains and may shed light on the reasons why initial cereal cultivation appeared with a high temporal variability throughout the Near East.

A further goal of this paper is to identify constraints in the currently available information on Near Eastern wild grass use and to evaluate how future research should address this issue to proceed in reconstructing the origin of charred grains in anthropogenic deposits.

## Material and methods

### The site of Chogha Golan and its palaeoecology

**Fig 1:**
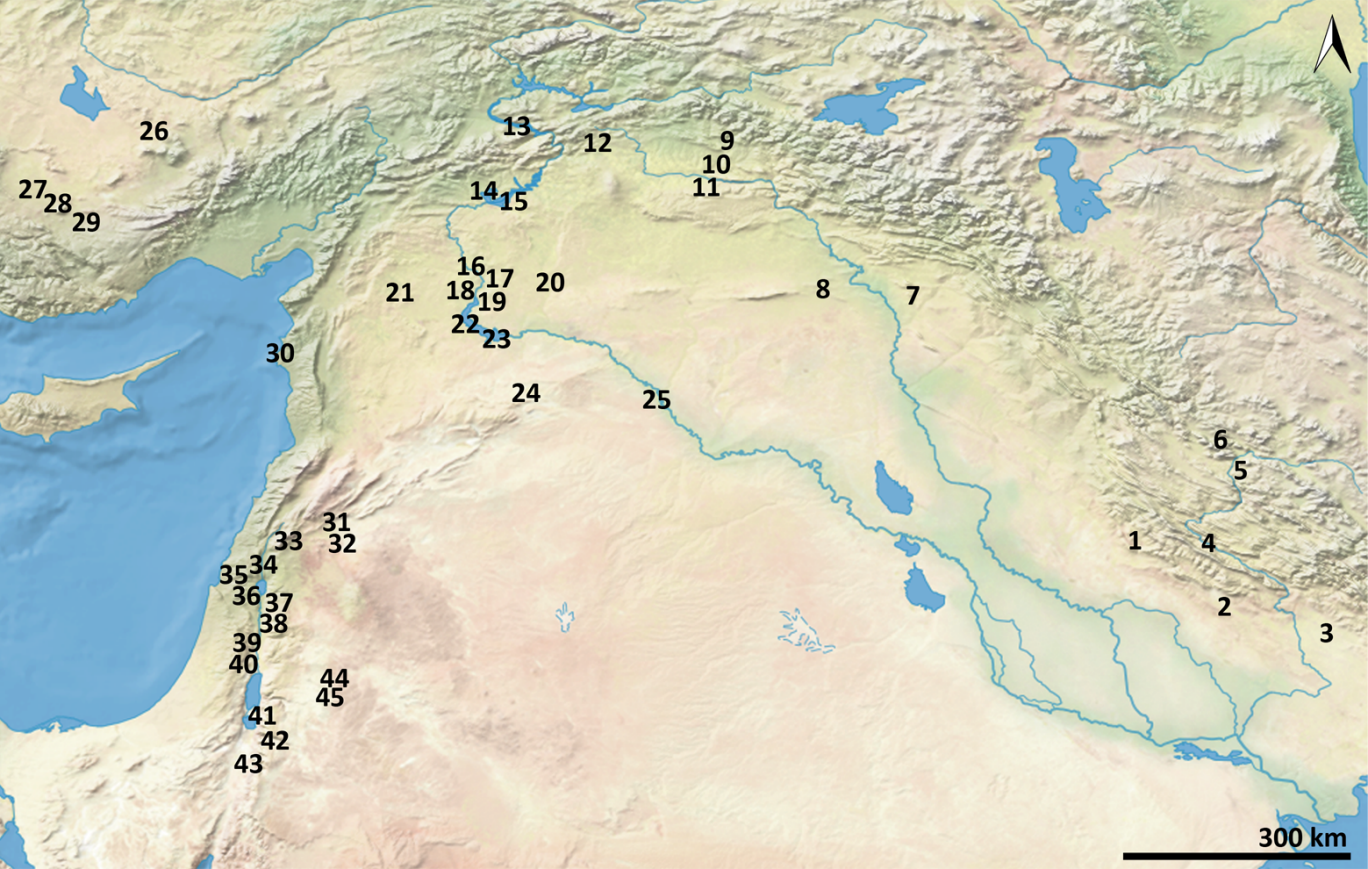
Epipalaeolithic and aceramic Neolithic sites mentioned in the text. (1) Chogha Golan; (2) Ali Kosh; (3) Chogha Bonut; (4) Chia Sabz; (5) Ganj Dareh; (6) Sheikh-e Abad; (7) M’lefaat; (8) Qermez Dere; (9) Hallan Çemi; (10) Demirköy; (11) Körtik Tepe; (12) Çayönü; (13) Cafer Höyük; (14) Gritille; (15) Nevali Çori; (16) Tell ‘Abr; (17) Dja’de; (18) Halula; (19) Jerf el Ahmar; (20) Sabi Abyad II; (21) Tell Qaramel; (22) Mureybet; (23) Abu Hureyra; (24) El Kowm II; (25) Tell Bouqras; (26) Aşikli Höyük; (27) Çatalhöyük; (28) Pinarbaşi; (29) Can Hasan III; (30) Ras Shamra; (31) Tell Ghoraifé; (32) Tell Aswad; (33) Tell Ramad; (34) Eynan; (35) Hilazon Tahtit; (36) Ohalo II; (37) Wadi al-Hammeh 27; (38) Iraq ed-Dubb; (39) Gilgal; (40) Netiv Hagdud; (41) ZAD 2; (42) el-Hemmeh; (43) Wadi Faynan 16; (44) Kharaneh IV; (45) Wadi el-Jilat 6 & 7.

The aceramic Neolithic site of Chogha Golan is located in the foothills of the Central Zagros Mountains in Iran (Fig 1). Members of the Tübingen Iranian Stone Age Research Project (TISARP) and the Iranian Center for Archaeological Research excavated the site in 2009 and 2010 [61]. The tell covers an area of about 3 hectares and is situated near the right bank of the Konjan Cham River (S1A Fig). Two trenches, a deep sounding and excavation area A, were opened in the center of the mound (S1B Fig). The deep sounding has an extent of 2 x 1.5 m and was excavated into sterile sediments 8 m below the tell surface. The excavators divided the stratigraphy of the deep sounding into eleven archaeological horizons (hereafter AH; S1C Fig). A series of radiocarbon dates places the occupation between ca. 11,700 and 9,600 cal. B.P. [16]. First evidences for substantial architecture, represented by remains of a mud-brick wall and a plaster floor, date to between ca. 11,000 and 10,600 cal. B.P., suggesting that permanent occupation at Chogha Golan lasted for at least 1,000 years or longer. The ground stone assemblage consists of mortars, pestles, grinding slabs, handstones, and pounders, while the chipped stone industry is characterized by bladelet production and little variance in tool types [62,63].

Riehl et al. presented first results on the palaeoecology of Chogha Golan [64]. According to the anthracological analysis, the nearby semiarid *Pistacia*-*Amygdalus* woodland steppe and the riparian vegetation were exploited for wood resources. The faunal material is dominated by ungulates and contains a high abundance of fish remains, but the preliminary results and the small sample size do not allow drawing final conclusions on possible management practices or morphological domestication [65]. The available archaeobotanical data indicate the exploitation of a high diversity of wild plant resources including a large component of wild progenitor species [16,44]. A considerable increase in small-seeded grasses between AH V and III may indicate a shift in resource availability with a possible contribution of climatic fluctuations. The abundant wild barley grain and chaff remains, combined with temporal grain size increases paralleled by high δ13C values indicative of enhanced growing conditions, may reflect management practices during this phase [64] and we do not rule out this possibility for the equally abundant *Aegilops* sp. remains [44]. Domestic-type emmer wheat chaff dominates the cereal remains of AH II and I and represents the first unequivocal evidence for the cultivation of a domestic crop at the site by ca. 9,800 cal. BP [16,27,44].

### Sampling and laboratory methods

For the present study the first author analyzed 23 additional flotation samples from AH VII to III of the deep sounding in order to increase the sample size for the occupation period that precedes the beginnings of agriculture at Chogha Golan and is contemporary to the early and middle PPNB of the Levantine sequence. A radiocarbon date from AH VIII dates to about 10,600 cal. BP and predates the sediments studied in this paper, for which radiocarbon dates accumulate between ca. 10,000 (AH VI-IV) and 9,800 (AH III-II) cal. BP [64]. Whether the age of AH VII is more close to 10,600 or 10,000 cal. BP is currently not clear. For illustrating the development of gathering and cultivation practices after the accumulation of these sediments, we use the data from 15 samples from AH II of the deep sounding. These have been analyzed by Weide et al., who showed that they are representative of the general subsistence economy during this final settlement phase [44].

The analyzed flotation samples derive from two main sediment types. Collapsed and decayed building debris intermixed with occupation deposits formed AHs II, III, V and VII, whereas AHs IV and VI represent midden deposits mostly consisting of ash, charcoal, bone fragments and stone artifacts [61]. Sediments macroscopically containing a high ash component and carbonized plant remains were sampled for bucket flotation using a 0.2 mm mesh. All laboratory work was conducted at the Institute for Archaeological Sciences at the University of Tuebingen, where the archaeobotanical material is currently archived. The botanical remains were identified using a Euromex binocular with 10 to 60x magnifications and the botanical comparative collection. For convenience, designation of species within the Triticeae follows the traditional classification [66]. Quantification methods followed the procedure described elsewhere [44]. We did not calculate ubiquity values for the taxa in the samples from the deep sounding, because most of them would have a very high frequency of up to 100% as an artifact of the small sampling area.

### Classification of the Poaceae taxa

Archaeobotanists traditionally divide Poaceae taxa into groups according to the dimensions of their caryopses. As an alternative approach we decided to use grain weight, based on the average 1000 seed weight given in the Seed Information Database of the Royal Botanic Gardens Kew [67]. Due to the variability and overlap in grain dimensions and weight, a strict division of taxa into size classes can hardly be achieved without additional criteria (S1 Table, S2 Fig). We therefore used the common functional interpretation of grasses alongside grain weight to form groups for our analyses (Table 1).

**Table 1:** Classification of Poaceae taxa frequently identified among Epipalaeolithic and aceramic Neolithic archaeobotanical assemblages from the Near East.

Wild and/or domestic cereals^1^	Large to medium-seeded wild grasses^2^	Small-seeded wild grasses^3^
*Hordeum spontaneum*/*distichum*	*Aegilops* spp.	*Aeluropus* spp.^4^
*Triticum boeoticum*/*monococcum*/*urartu*	*Avena* spp.	*Alopecurus* spp.
*Triticum dicoccoides*/*dicoccum*	*Bromus* spp.	*Agrostis* spp.^4^
	*Echinaria capitata*	*Crypsis* spp.^4^
	*Eremopyrum* spp.	*Echinochloa* spp.
	*Hordeum* spp. (without *spontaneum*)	*Eragrostis* spp.^4^
	*Lolium* spp.	*Phalaris* spp.
	*Piptatherum* spp. (only the larger	*Phleum* spp.
	*holciforme*-type has been identified)	*Poa* spp.^4^
	*Secale* spp.	*Puccinellia* spp.^4^
	*Stipa* spp.	*Setaria* spp.
	*Taeniatherum caput-medusae*	*Sporobolus* spp.^4^

^1^ Wild progenitor species and their domesticated relatives; commonly interpreted as food plants

^2^ Wild grasses with an average 1000 seed weight of 3g or more that do not belong to the founder crops; commonly interpreted as food plants and/or weeds

^3^ Wild grasses with an average 1000 seed weight less than 3g; high proportions are often interpreted as representing dung-burning or fuel collection

^4^ These taxa have been interpreted as fuel or dung-derived [18,39,57,85,87,90].

The “small-seeded wild grasses” comprise genera with an average grain weight of less than 3g. Many species in this group are commonly interpreted as representative of dung-burning or fuel collection activities. From this group we separate all genera with an average grain weight of 3g or more. These taxa are commonly regarded as potential food plants or arable weeds and most of them have a reduced probability of being preserved in herbivore dung due to their larger grain size [18,68,69]. We further differentiate among these taxa between grasses that were domesticated during the aceramic Neolithic and were thus often interpreted as pre-domestic cultivars, and those that are no progenitors of the classical founder crops [70]. We refer to this second group as “large to medium-seeded wild grasses”, whereas the first group contains the “wild and domestic cereals”. A possible classification of some large-seeded wild grasses such as *Avena sterilis* or *Aegilops* spp. as cereals will be discussed below. In this regard it is important to note that the functional interpretations assigned to taxa within a group are not mandatory and single taxa can still be interpreted differently. Combining different criteria based on physiological characteristics of grains and possible usage currently provides the most plausible, knowledge-based classification for our analyses.

### Correspondence analysis

Correspondence analysis is a multivariate statistical method routinely used by archaeologists to examine the complex relationships between variables in a contingency table [71]. In our analysis the variables represent the botanical taxa identified from the charred plant remains, whereas the cases represent the flotation samples. We applied correspondence analysis to investigate how the samples from AH VII-II of the deep sounding differ in their taxonomic composition and whether these differences can be attributed to the chronological order of the horizons or the sediment types.

The original dataset included 93 taxa in 38 samples. To avoid the bias of rare species and a poor preservation, we omitted all taxa with a ubiquity lower than 10% and all samples containing less than 100 identified specimens from the analysis. Because this procedure would have substantially influenced the original dataset, we combined rare taxa with a similar taxonomic rank or ecological implication in order to reach a ubiquity of 10%. Following this procedure we could maintain the majority of the original data and generated a table comprising 47 taxa in 36 samples.

Multivariate statistics can be used to disentangle cultivated species, arable weeds, gathered resources and dung-derived plant remains, given a large dataset deriving from a multitude of contexts is available (see e.g. [18]). As the majority of samples from Chogha Golan derives from the relatively narrow deep sounding, only representing one context per settlement phase, we could unfortunately not apply a multivariate approach to disentangle the botanical taxa in this regard.

### A temporal and spatial analysis of Near Eastern wild grass use

We systematically reviewed the published archaeobotanical literature for examining wild grass use throughout the Epipalaeolithic and aceramic Neolithic. After screening all published reports, we defined standard criteria for subsequent analyses, which resulted in differing sets of sites for the conducted analyses and allowed to consider a maximum number of datasets for reviewing wild grass use (S3 Fig). A total of 14 individual archaeobotanical assemblages were excluded, because the plant remains were too poorly preserved or not fully published. Assemblages deriving from only one single context, e.g. the grain storage from Gilgal I, were also omitted from the quantitative analyses.

For our analyses we used the proportions of the different Poaceae taxa and categories together with their ubiquity values. Taphonomic processes and diverging quantification methods can result in a heavy overrepresentation of single botanical taxa in charred assemblages. For instance, several PPNA sites in the southern Levant yielded extremely high counts for fig nutlets and *Pistacia* shell fragments, which resulted in a strong proportional decrease of all other plant groups [28,35,72]. To avoid such biases in our interpretations, we did not plot the proportions of the grass categories together with all other plant remains. Instead, we only analyzed the proportions of the different grass categories for all sites that yielded more than 100 caryopses, regardless of the analyzed sample number. Furthermore, we excluded Poaceae chaff remains and indeterminate cereal grain fragments from this analysis. Chaff of non-cereal taxa is rarely preserved in charred archaeobotanical assemblages, which often contain high proportions of identifiable cereal chaff. This disproportion in the occurrence of spikelet remains from different groups would substantially bias our results towards decreased percentages for non-cereal taxa. Similarly, indeterminate cereal grain fragments are often very abundant and included in the published data. Their numbers are rather indicative of fragmentation rates and regularly result in extremely high proportions of the cereal grain category. Therefore, we only compared the identifiable cereal grains with the number of caryopses from non-cereal taxa, which rarely contain high counts of indeterminate fragments. This procedure gave consistent results and effectively reduced the influence of different survival ranges or quantification methods.

Ubiquity values were used to plot the occurrence and frequency of selected genera over time. We defined a threshold of ten flotation samples to include sites into this analysis. Different occupation phases have occasionally been combined in order to reach this minimum number of samples. For Chogha Golan we combined the samples from the deep sounding with those from excavation area A [44] to obtain unbiased frequencies. Ten samples is a quite low number to calculate ubiquity values, but as we can show below, it proved sufficient to investigate wild grass abundance over time. In addition, we included all sites with more than ten analyzed flotation samples into this analysis, from which ubiquity values could not be calculated based on the way the data were published. For these sites we only indicated the presence or absence of taxa. Interestingly, such publications accumulate in the Epipalaeolithic and PPNA. Excluding them would have resulted in a biased occurrence of wild grasses for these periods. Finally we have to clarify that, for practical reasons, we only use the term Pre-Pottery Neolithic and its subdivisions into PPNA, early, middle and late PPNB in a strict chronological and not in a cultural sense.

## Results

### The composition of the archaeobotanical material from Chogha Golan

S2 Table gives the plant remains from the samples analyzed for this study. The assemblage comprises 81 taxa from 20 families, summing up to about 23,500 items. The midden deposits of AH IV and VI provided the richest samples with an average find density of 195 items/liter soil and a maximum of 360 items/liter soil in a sample from AH IV. Samples from the decayed building debris had lower find densities with an average of 55 items/liter soil and a maximum of 147 items/liter soil in AH III.

#### The Poaceae remains

**Fig 2:**
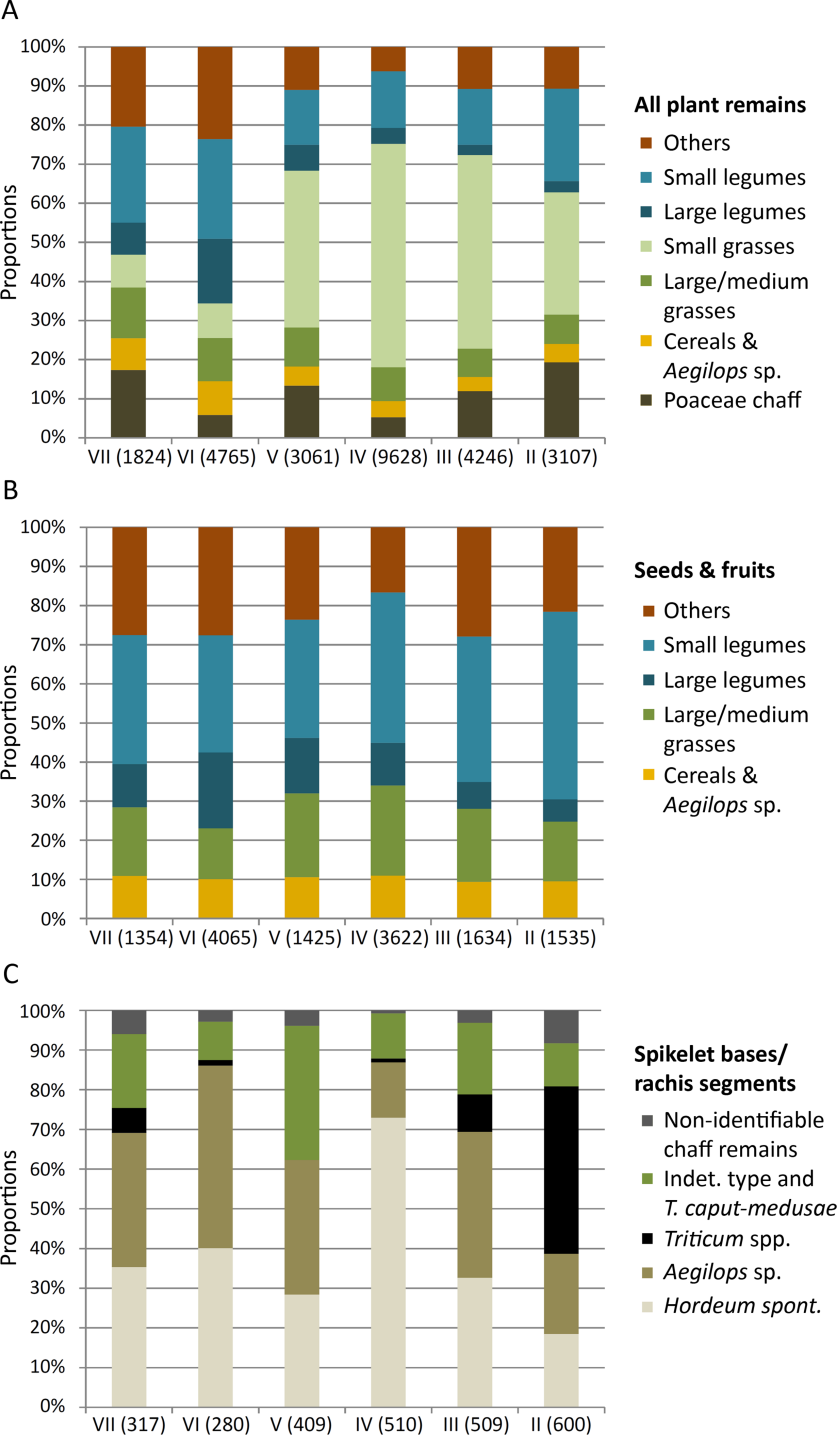
Proportional development of the major taxonomic groups from AH VII to II in the deep sounding of Chogha Golan. (A) Proportions of all analyzed plant remains; (B) proportions of all seeds and fruits without the small-seeded grasses; (C) proportions of Poaceae chaff only. Numbers in brackets give absolute counts.

Poaceae grains and chaff represent the most abundant plant remains in the analyzed samples with overall proportions between 34% in AH VI and 75% in AH IV (Fig 2A). The small-seeded taxa *Phalaris* sp. and *Phleum* type, together with small indeterminate grains, contribute the major portion to the grass remains. Large to medium-seeded taxa such as *Avena* sp., *Bromus* sp., *Hordeum* sp., *Eremopyrum* sp., *Taeniatherum caput-medusae* and Triticoid type form a second abundant group. They are mostly represented by grains and yielded only few identifiable chaff remains. Along with fragmented spikelet bases of *T*. *caput-medusae*, spikelets of an indeterminate type have been frequently found. Among the cereal species, *Hordeum spontaneum* is well represented by both grains and rachis segments. The domestic-type rachises reach their highest proportion in AH VII with 3.6%, still indicative of a population with shattering ears. Species of *Triticum* only yielded glume bases and spikelet forks, but virtually no identifiable grains. The major portion of the identifiable rachis and glume base fragments could be attributed to emmer wheat, whereas einkorn constitutes a minor component of the wheat chaff. The emmer chaff from AH II was identified as phenotypically domesticated [16,27,44], whereas the chaff remains from the older horizons mostly contain non-diagnostic specimens. *Aegilops* sp. grains and chaff yielded proportions comparable to these typical cereal species. We therefore regard *Aegilops* sp. as economically equally important as *H*. *spontaneum* and the *Triticum* species and include it into the cereal category at Chogha Golan (but see the discussion below).

Since the cereals yielded such abundant identifiable chaff remains, but the other wild grass taxa did not, we excluded the Poaceae chaff to evaluate the relative proportions between cereals and the large to medium-seeded wild grasses. Here, the small-seeded grasses have also been omitted, because their high percentages have a large impact on the relative proportions of all other taxonomic categories. Excluding these two groups allowed interpreting the proportional development of the cereals and large to medium-seeded wild grasses more directly (Fig 2B). Now, grains of *H*. *spontaneum* and *Aegilops* sp. continuously make up about 10% of the assemblage, whereas the other large to medium-seeded wild grasses show a gradual development throughout the analyzed sequence. They increase from AH VII (18%) to AH IV (23%) and again decrease towards AH II (15%). In their entirety, these large to medium-sized grains constitute higher proportions to the analyzed material than cereal grains. However, the lower grain volumes of the medium-seeded grasses have to be taken into account while assessing the relative importance of these taxa compared the cereals (see S2 Fig).

Fig 2C gives the relative proportions of the analyzed chaff remains. Rachises and spikelet bases of *H*. *spontaneum* and *Aegilops* sp. dominate AH VII to III, where they contribute between 62% and 87% to all chaff remains. In these horizons they are equally abundant except for AH IV, where *H*. *spontaneum* rachises outnumber *Aegilops* spikelets by far. This trend is also visible for the grains and might represent a bias due to unknown factors that contributed to the formation of these midden deposits. *Triticum* chaff has relatively low values between AH VII and III, but substantially increases towards AH II where it dominates the chaff remains (42%). The spikelets of the indeterminate type and *Taeniatherum caput-medusae* fluctuate throughout the sequence (10–34%) and only reach proportions comparable to the cereal species in AH V.

**Fig 3:**
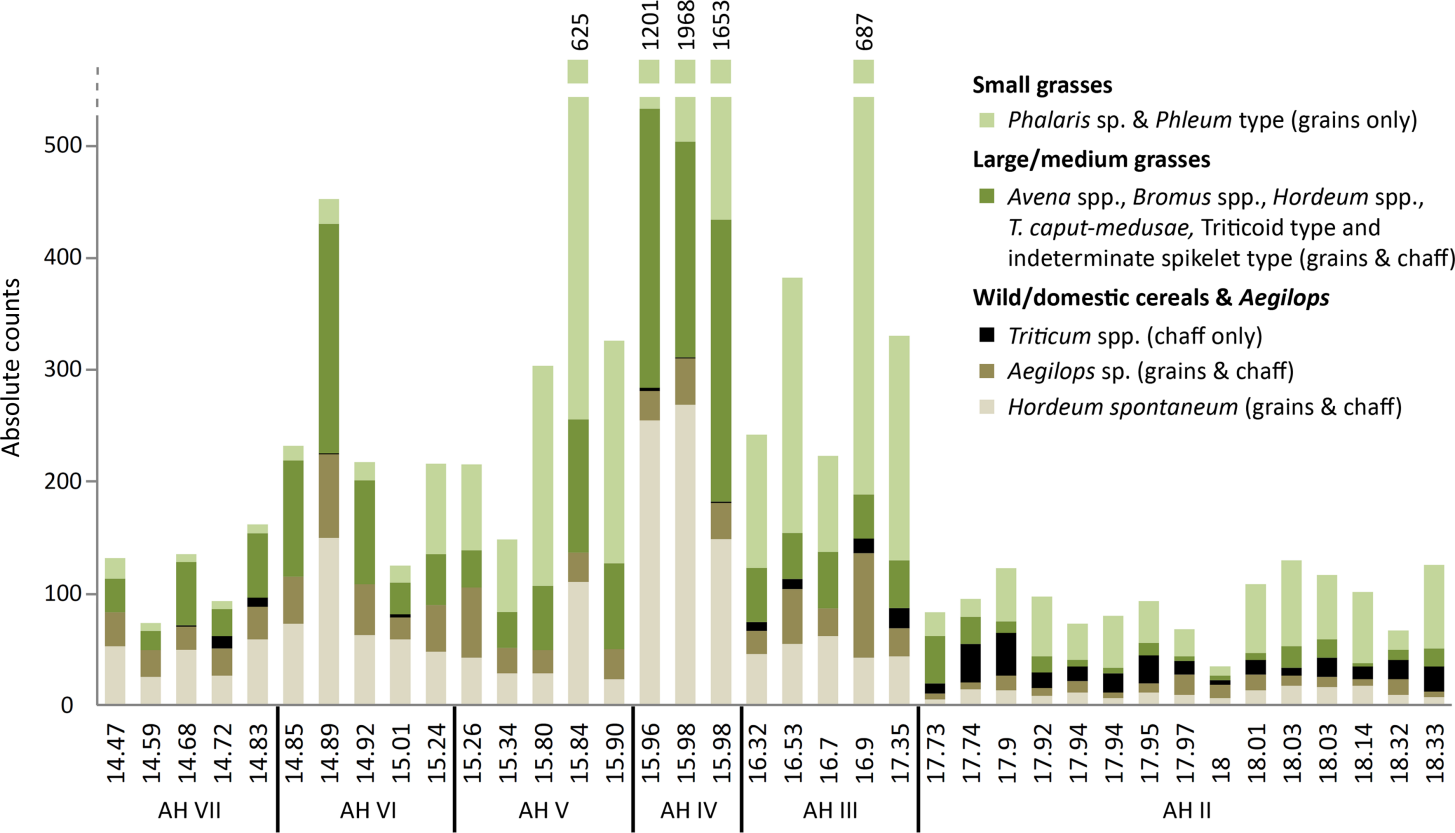
Composition of identifiable Poaceae remains in samples from AH VII to II in the deep sounding of Chogha Golan. Note that the categories are represented by different plant organs. The x-axis gives z-values of the deep sounding starting with 20m at the tell surface; the y-axis gives absolute counts up to 500, higher counts are given above the respective bars.

Although most grass taxa are solely represented by grains and *Triticum* spp. only yielded chaff, the temporal development of the different grasses becomes apparent by plotting all these data together (Fig 3). Despite the paucity of identifiable chaff remains among the large to medium-seeded wild grasses, they reach counts as high or higher as the cereals between AH VII and IV, indicating an important role in the subsistence of Chogha Golan. With the appearance of *Triticum* spp. chaff in AH III and its dominating status in AH II, all wild grasses together with *H*. *spontaneum* and *Aegilops* sp. decrease in absolute and relative numbers.

#### Fruits and seeds of other plant groups

Legume seeds represent the second largest group of plant remains in the analyzed samples. Large legumes such as *Lens* sp., *Pisum* sp. and species of *Lathyrus* and *Vicia* contribute about 17% to AH VI, but range between 3% to 8% in all other horizons. These values are only slightly smaller than the proportions for the cereal grains, which remain constant while the large legume seeds steadily decline towards AH II (Fig 2A and 2B). In contrast, small legume seeds belonging to the genera *Astragalus*, *Trigonella* and *Medicago* reach percentages between 14% and 25% (Fig 2A). The development of their relative abundance is significantly affected when plotted together with the small-seeded grasses and the Poaceae chaff. Then they seem to decline from the older horizons towards AH III, but show rather constant percentages after the small grasses and chaff remains are excluded (Fig 2B).

In addition to the grasses and legumes, between 6% and 24% of the assemblages per horizon consist of fruits and seeds of other wild taxa (Fig 2A; S2 Table). After excluding the small grasses and chaff remains, they have fluctuating percentages between 17% and 28%. Among them, edible fruits and seeds of *Atriplex* sp., *Bolboschoenus glaucus*, *Malva* sp. and several crucifers (*Alyssum* sp., *Capsella*/*Descurainia*, *Lepidium* sp.) abundantly occur in the analyzed samples. Particularly *Atriplex* sp. and *Alyssum* sp. seeds reach high counts in the samples from the midden deposits of AH VI. Nutshell fragments of *Pistacia* represent the only remains of a gathered tree-fruit. They constantly occur in all samples, but are significantly less abundant in the middens of AH VI and IV (S2 Table).

### Major factors influencing sample composition at Chogha Golan

**Fig 4:**
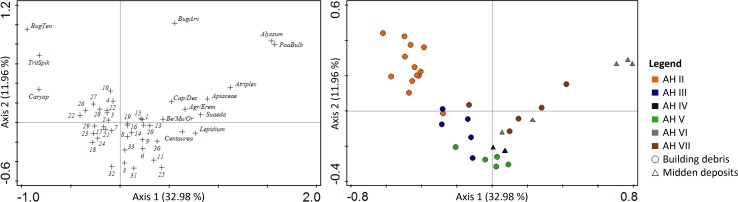
Scatter plots of a correspondence analysis testing for inter-sample variation in the deep sounding. The plots show the distribution of (A) 47 taxa/categories from (B) 36 flotation samples. See S3 Table for coding of the variables and S4 Fig for the composition of Poaceae remains in the samples used in the correspondence analysis.

The correspondence analysis plot in Fig 4 shows a sample distribution that follows a parabola, which is also known as “horseshoe-effect” [71]. Samples from AH II are placed at the left end of the first axis and are clearly separated from samples of the underlying horizons III to VII along axis one. Chaff of *Triticum* spp. together with seeds from the Caryophyllaceae family and uncarbonized *Buglossoides tenuiflora* nutlets are the most important variables that induce this separation. Among the other horizons, AH III is separated from AH IV and V along the first axis. These two horizons completely overlap and are again separated from the oldest horizons VI and VII. Three samples from AH VI form a distinct group at the right end of axis one. This is particularly due to a high proportion of *Alyssum* sp. and *Atriplex* sp. seeds and bulbils of *Poa bulbosa* in these midden-derived samples. Only here the taxonomic composition of the samples is significantly influenced by the type of deposit, whereas all other samples follow a strong chronological trend throughout the stratigraphy. The overall composition of Poaceae remains also follows this temporal trend (S4 Fig).

### Wild grasses in the Epipalaeolithic and aceramic Neolithic of the Near East

#### Grain proportions, single contexts and author’s interpretations

**Fig 5:**
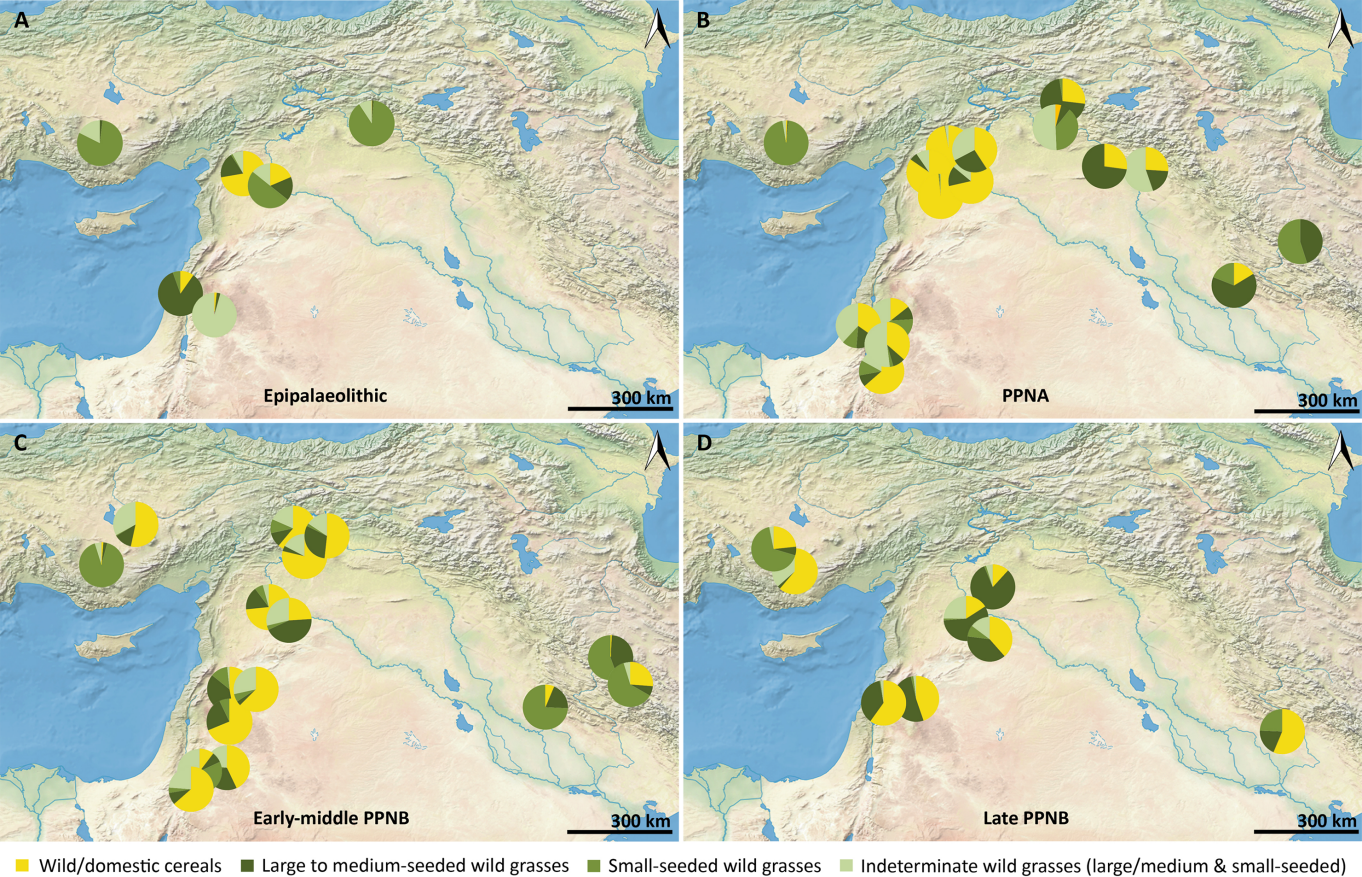
The composition of Poaceae grains in archaeobotanical assemblages dating to the Epipalaeolithic and Pre-Pottery Neolithic of the Near East. See S5 Fig for a detailed key to the sites represented by the pie charts.

Fig 5 shows the proportions of four groups within the Poaceae grains identified at Epipalaeolithic to late PPNB sites of the Near East. References for all investigated sites including their general chronological order and basic information about the author’s interpretations of the wild grass remains are given in Table 2. From 72 published archaeobotanical datasets including distinct sub-phases of several sites, we used 46 datasets that yielded more than 100 Poaceae grains.

**Table 2:** The sites included in the quantitative analyses of this study with information on the number of samples, mesh size used during water flotation and the interpretation of the wild grass finds by the respective authors. Periods follow the Levantine sequence and have no strict cultural implications.

Site (phase or area)	C-14 dates (ka cal. BP;	Samples or	Mesh size	Author(s) interpretation(s)	Contextual evidence	References for botanical
	based on individual site	loci (n)	(mm)	of wild grasses*	for wild grass* use/origin	data and interpretations
	reports and [133,134])					
**Epipalaeolithic**						
Ohalo II	23	unknown	unknown**	Staple food/arable weeds	*in situ* working area	[38,49,73,74]
Pinarbaşi (Epipalaeolithic)	16.2–12.9	25	0.1	Fuel, construction materials		[75]
Wadi al-Hammeh 27	14–13.7	14^Ub^	0.3	Potential food		[40]
Wadi el-Jilat 6	13.7	10^Ub^	0.3	Potential food		[40]
Abu Hureyra (1)	13.2–12.8	unknown	1	Potential food/arable weeds		[121]
Körtik Tepe (Younger	12.4–11.7	116^Ub^	0.2	Staple food		[60]
Dryas)						
**Epipalaeolithic/PPNA**						
Tell Qaramel (H2)	12.3–11.3	108 (H1-3)	0.5	Harvested accidentally with	*Stipa* sp. grains concentrated	[76]
				wild cereals	in one sample	
Mureybet (I-II)	12.2–11.3	33^Ub^	unknown	Potential food		[24]
Qermez Dere	12.1–10.8	47^Ub^	0.35	Staple food		[51,130]
**PPNA**						
Sheikh-e Abad (Trench 1)	11.8–11.2	5	0.25	Potential food and dung	Separated in CA from crop/	[18]
				burning (esp. *Poa* type 1)	weed group	
Hallan Çemi	11.7–11.3	175^Ub^	0.35	Potential food		[51,130]
M'lefaat	11.7–10.8	4	0.35	Staple food		[51,130]
Körtik Tepe (Early	11.6–11.3	231^Ub^	0.2	Staple food		[60]
Holocene)						
Chogha Golan (XI-VIII)	11.7–10.6	13	0.2	Undetermined		[16]
Iraq ed-Dubb (structures)	11.5–11.3	7	0.3	Potential food/arable weeds		[40]
Jerf el Ahmar	11.5–10.6	266^Ub^	0.5	Potential food/arable weeds	Context with concentration of	[32]
					*Aegilops* sp. spikelet bases	
Demirköy	11.4–11.3	12^Ub^	0.35	Potential food		[51,130]
Mureybet (III)	11.4–10.5	28^Ub^	unknown	Potential food		[24]
Netiv Hagdud	11.3–10.9	58	0.5	Potential food		[28]
Tell Qaramel (H3)	11.3–10.9	108 (H1-3)	0.5	Harvested accidentally with		[76]
				wild cereals		
Tell 'Abr	11.3–10.9	30^Ub^	0.5	Arable weeds		[32]
el-Hemmeh	11.2–10.6	15 loci^Ub^	0.25	Potential food/arable weeds		[35,80]
ZAD 2 (Structure 1)	11.1–10.8	18 loci^Ub^	0.5	Subsistence *sensu lato*		[33,72]
Pinarbaşi (early)	11–10.6	13^Ub^	0.1	Potential food or fuel		[57]
**PPNA/early PPNB**						
Wadi Faynan 16	11.4–10.3	44	0.25	Potential food		[131]
Tell Aswad (I)	11.3–10.4	9	unknown	Arable weeds		[23]
Dja'de	11.1–10.3	227^Ub^	0.5	Potential food/arable weeds		[32]
**early PPNB**						
ZAD 2 (Structure 2)	10.7–10.5	8 loci	0.5	Subsistence *sensu lato*		[72]
Tell Qarassa (Area XYZ)	10.7–10.2	58^Ub^	0.25	Arable weeds/undetermined		[81]
Çayönü (grill-channel)	10.6–10.2	96^Ub^	unknown	Arable weeds/undetermined		[79]
**early/middle PPNB**						
Tell Aswad (II)	10.6–9.5	21^Ub^	unknown	Arable weeds		[23]
Pinarbaşi (late)	10.6–9.7	27^Ub^	0.1	Potential food or fuel		[57]
Nevali Çori	10.5–9.6	267	0.35	Arable weeds/undetermined		[94]
Cafer Höyük	10.3–9.5	62^Ub^	0.3	Undetermined		[42]
Aşikli Höyük	10.3–9.4	144^Ub^	unknown	Undetermined		[82]
**middle PPNB**						
Chogha Golan (VII-II)	10.2–9.8	98^Ub^	0.2	Staple food		This paper and [44]
Ganj Dareh	10–9.5	122^Ub^	unknown**	Potential food/undetermined		[39]
Sheikh-e Abad (Trench 2)	10	9	0.25	Potential food and dung	Separated in CA from crop/	[18]
				burning (esp. *Poa* type 1)	weed group	
el-Hemmeh	9.8–9.6	31 loci^Ub^	0.25	Arable weeds/animal fodder		[80]
Sheikh-e Abad (Trench 3)	9.6	27^Ub^	0.25	Potential food and dung	*Poa* type 1 constitutes over	[18]
				burning (esp. *Poa* type 1)	90% to the samples	
**middle/late PPNB**						
Çayönü (cobble-cell)	10.3–9.4	105^Ub^	unknown	Arable weeds/unspecified		[79]
Abu Hurerya (2A)	9.8–9.4	41^Ub^	1	Potential food/arable weeds		[42]
Tell Halula	9.8–9	96	0.2	Potential food (*Aegilops*)/		[108]
				arable weeds		
Wadi el-Jilat 7 (Middle)	9.7–9.4	21^Ub^	0.3	Potential food/arable weeds		[40]
Tell Ghoraifé (I)	9.9–9.4	18^Ub^	unknown	Arable weeds		[23]
**late PPNB**						
Ali Kosh (BM-AK)	9.5–9	unknown	unknown	Arable weeds/wild resources		[21]
Sabi Abyad II	9.5–8.8	10	unknown	Arable weeds		[89]
Ras Shamra (Vc)	9.5–9.1	25^Ub^	unknown	Arable weeds		[95]
Gritille	9.5–8.7	52^Ub^	1	Undetermined		[132]
Can Hasan III	9.5–8.5	4	1	Arable weeds/undetermined		[83,129]
Abu Hurerya (2B)	9.4–9.1	43^Ub^	1	Potential food/arable weeds		[42]
Tell Bouqras (Sq. 16/13)	9.4–8.2	97^Ub^	unknown	Undetermined		[96]
Tell Ramad (I)	9.3–9	13^Ub^	unknown	Arable weeds		[23]
Tell Ghoraifé (II)	9.1	17^Ub^	unknown	Arable weeds		[23]
Çatalhöyük East (VI-PXII)	9.1–8.5	61^Ub^	0.3	Potential food/arable weeds/	Storage finds of *Taeniatherum*	[41,53,87,88]
				dung burning, basketry	*caput-medusae* grains	
El Kowm (II)	9–8.5	31^Ub^	0.3	Arable weeds/dung burning		[42]

^Ub^ ubiquity was given in/calculated based on publication

* regardless of the wild cereals, which might be interpreted differently

** no information available, but small-seeded grasses were recovered in considerable abundance

CA = correspondence analysis

The data from the Epipalaeolithic comprise six sites that span a relatively large time period from ca. 23,000 to the 12^th^ millennium cal. BP (Fig 5A). Ohalo II is the oldest of these sites and contains, like Wadi al-Hammeh 27 and Körtik Tepe, a minor proportion of wild cereal grains. These sites are dominated by large to medium-seeded wild grasses, indeterminate specimens and small-seeded taxa, respectively. Despite these differences in the dominant groups, wild grasses have been regarded as important food resources at all three sites, possibly representing staples at Ohalo II and Körtik Tepe [40,49,60,73,74]. Wild progenitors are completely absent at Pinarbaşi, where small-seeded taxa were possibly gathered for fuel or construction materials [75]. In contrast, horizon 2 at Qaramel and the early phases at Mureybet date to the Pleistocene-Holocene transition and already show increased values for the cereal group. Non-cereal taxa including *Eremopyrum* sp., *Echinochloa* sp. and a type resembling *Setaria* still make up the major portion of grains at Mureybet and were regarded as possible food resources [24]. At Qaramel cereal grains clearly dominate the assemblage and seem to represent the earliest evidence for such a strong focus on wild progenitor species, in this case einkorn and barley. Willcox and Herveux regarded the large to medium-sized grains, of which the far majority comes from one single sample and was identified as *Stipa* sp., as a possible contaminant of the wild cereal harvests [76].

During the PPNA wild cereals became the dominating taxa at sites in the Levantine corridor (Fig 5B). This development was associated with the emergence of potential arable weed floras, a gradual decrease of formerly gathered species and an increase in cereal grain size at sites in the upper Euphrates region. Willcox et al. interpreted this pattern as the beginnings of wild cereal cultivation at Jerf el Ahmar, Qaramel, Dja’de and ‘Abr [32]. Colledge explored the data from Mureybet with multivariate statistics and came to the same conclusion [30], as already hypothesized by van Zeist and Bakker-Heeres [24]. Large to medium-seeded wild taxa are not numerous at these sites except for Jerf el Ahmar and Dja’de. Willcox et al. regarded them as potential arable weeds, but also noted that they represent potentially edible resources [32]. At Jerf el Ahmar, *Hordeum murinum*/*bulbosum* grains have very high counts and a single concentration of *Aegilops* spikelet bases possibly represents the remains of threshing. For Dja’de, grains of *Taeniatherum caput-medusae* reach counts comparable to the wild cereals. These patterns possibly indicate deliberate gathering of several wild grasses alongside early cereal cultivation in the upper Euphrates region. However, as the authors pointed out, their lower overall grain volume speaks against an economic role comparable to the cereals.

Although the assemblages from the southern Levant show a less marked dominance of wild cereal grains, pre-domestic cultivation has been proposed for all sites included in this analysis [28,35,40,72]. Identifiable large to small-seeded wild grasses represent a minor component of the grains from these sites. However, the major portion of the non-cereal grains from most sites remained unidentified, but would significantly add up to the percentages of identified specimens. Except for ZAD 2, where Meadows did not explicitly discuss these non-cereal taxa [72], all authors regarded them as possible food sources. Particularly for Netiv Hagdud and Iraq ed-Dubb, species of *Avena* or *Phalaris* were possibly exploited alongside wild cereals [28,40]. However, with the proposition of pre-domestication cultivation for many PPNA sites, scholars increasingly view crop-processing activities as possible explanations for the occurrence of these wild grasses in archaeobotanical assemblages. For instance, White and Makarewicz listed *Bromus* sp., *Hordeum glaucum* and *Phalaris* sp. as potential arable weeds, although they admitted that “there are many distinct plant exploitation strategies that could each produce an archaeobotanical assemblage exhibiting a weedy botanical spectrum (…)” [77]. Following this, they regarded the potential weed taxa as resources that were possibly harvested from the fields as a minor food source alongside the cultivated wild cereals. Based on similar considerations, Colledge did also not strictly distinguish between the non-cereal taxa as possible weeds or collected foods during the PPNA phase of Iraq ed-Dubb [40]. In contrast, grains and chaff remains of *Avena sterilis* associated with *Hordeum spontaneum* in a storage context at Gilgal clearly indicate that some large to medium-seeded wild grasses did represent important foods in the Levantine PPNA [78]. Weiss et al. even raised the possibility that this finding represents a harvest from cultivated fields [52], which would attribute wild oats a status as early cultivars.

Outside the Levantine corridor, evidence for an increased focus on the wild progenitor species during the PPNA does not exist. For central Anatolia we are currently left with only one site, Pinarbaşi, where wild cereals are virtually absent. The only abundant grass remains are charred culm nodes and the small grains of a *Puccinellia* species, which Fairbairn et al. interpreted as a possible fuel source [57]. In contrast, many sites in the Zagros and Taurus Mountains and the adjacent lowlands provide good evidence for diverse wild grasses gathered as food sources during the PPNA, with large to medium-seeded taxa being more abundant than the wild cereals. Whereas at Hallan Çemi and Demirköy the gathered grasses did not contribute a large portion to the whole charred assemblage and might represent a minor food resource, they are regarded as major foods and even staples at all other sites of this region included in the present analysis [18,51,60,64]. Pre-domestication cultivation was recently proposed for Körtik Tepe and Chogha Golan [16,60,64]. However, on both sites the wild progenitor grains are less abundant than the entirety of non-cereal taxa.

The overall pattern that was characteristic for the PPNA continues into the early and middle PPNB, where domestic cereals provide unequivocal evidence for the beginnings of farming [6,25]. Sites in the Levantine corridor display a clear focus on cereals, now including domestic species (Fig 5C). Large to medium-seeded wild grasses make up considerable proportions of many assemblages and were much more confidently interpreted as arable weeds [23,79–81]. Sites where the abundant non-cereal taxa were still regarded as possible foods are Abu Hureyra and Wadi el-Jilat 7, but also here domestic cereals are present and the wild grasses could represent weeds [40,42].

Such a clear focus on a cereal-based subsistence strategy continues to be rare outside the Levantine corridor in the early and middle PPNB. Aşikli Höyük represents the only site in central Anatolia where domestic cereals were cultivated [25,82]. Recent analyses indicate that wild grasses are still frequent at the site (M. Ergun, pers. communication) and we should wait for the final publication of the new results. In contrast, Pinarbaşi represents a presumably sedentary hunter-gatherer community, indicating the co-occurrence of distinct subsistence economies during the local establishment of agriculture [57]. In the central Zagros Mountains, plant domestication was proposed based on domestic-type emmer chaff for Chogha Golan and on domestic-type barley grains for Sheikh-e Abad and Ganj Dareh [16,18,27,39,44]. As shown above, large to medium-seeded wild grasses represent major foods at Chogha Golan and decrease with the emergence of domestic emmer. At Sheikh-e Abad wild grasses are also abundant in the levels dating to the middle PPNB, whereas they are outnumbered by barley grains at Ganj Dareh. Whitlam interpreted the non-cereal taxa at Sheikh-e Abad as gathered wild foods based on the outcome of correspondence analyses [18]. In her plots, the large to medium-seeded wild grasses are separated from a crop-weed-group comprising the domestic-type barley grains and potential arable weeds.

Full agricultural societies are established in all regions of the Fertile Crescent with the late PPNB (Fig 5D). The archaeobotanical record for central Anatolia and the central Zagros is again very poor and the few investigated sites represent typical farming villages with a clear focus on crop cultivation [21,41,83]. Dung burning seems to have played a critical role in the formation of the charred plant assemblages from Çatalhöyük and Ali Kosh and dung pellets were found during both excavations [41,84,85]. Fairbairn and his colleagues used Pearson correlations for associating the potential weeds with cereal grains and chaff at Çatalhöyük. Among the analyzed taxa, *Eremopyrum*-type and *Stipa* sp. grains positively and significantly correlated with cereal grains, supporting their interpretation as common field weeds. Interestingly, grains of *Taeniatherum caput-medusae* did not show a significant correlation, but were found in storage contexts together with *Eremopyrum*-type grains [53]. The authors regarded these edible grains as potential resources, which were possibly utilized after crop procession rather than being disposed. In applying standard criteria for the recognition of dung-derived plant material in archaeobotanical assemblages [86] and correspondence analysis, Filipović confirmed the state of *Taeniatherum* and *Eremopyrum* as arable weeds at the site [87]. Grasses have also been gathered for other purposes, incl. basketry, which was demonstrated via phytolith analyses [88].

A remarkable development in the Levantine corridor, particularly the upper Euphrates area, is the increase in non-cereal taxa towards the late PPNB. Whereas de Moulins did not strictly rule out continuing gathering activities at Abu Hureyra [42], this pattern clearly reflects the establishment of some grass species as successful arable weeds at sites such as Ghoraifé, Ramad, El Kowm and Sabi Abyad II [23,42,89]. *Lolium* sp. represents the most common weed at these sites, which is accompanied by *Aegilops* sp. at Sabi Abyad II and by *Bromus* sp. at El Kowm.

#### Patterns in the record of the small-seeded grasses

**Fig 6:**
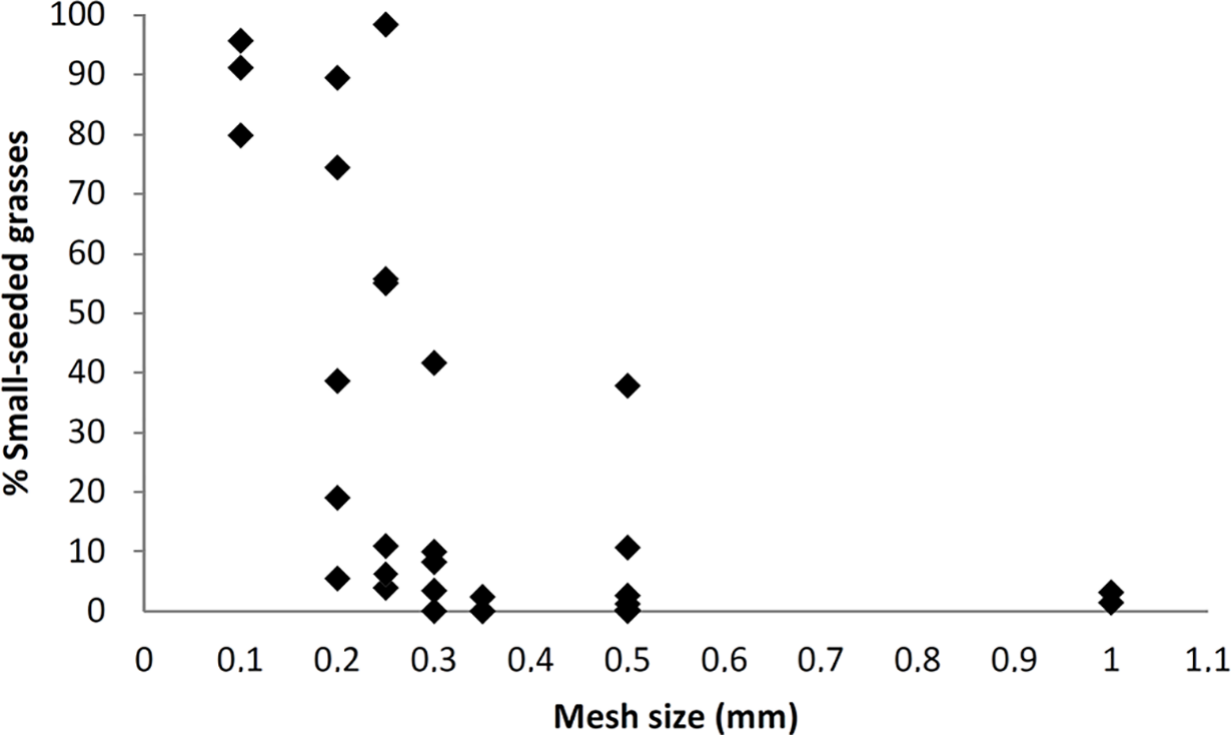
The proportions of small-seeded grasses among all Poaceae grains in relation to the mesh size used during water flotation.

Fig 6 shows the proportions of small-seeded grasses among Poaceae grains in relation to the mesh size applied during water flotation. This analysis includes 32 assemblages that yielded more than 100 grains. We found a decrease in the abundance of small-sized caryopses at sites where sieves had mesh sizes of > 0.25mm. The absence or scarcity of small-seeded taxa at these sites might therefore be biased by the applied field methods. However, the proportions of small caryopses can also be very low despite the application of fine sieves. At sites where sieves had mesh sizes of 0.25mm or less, the percentage of small-seeded grasses among all Poaceae grains varies between 0 and almost 100%. This suggests taphonomic rather than methodological factors for explaining the observed variability.

**Fig 7:**
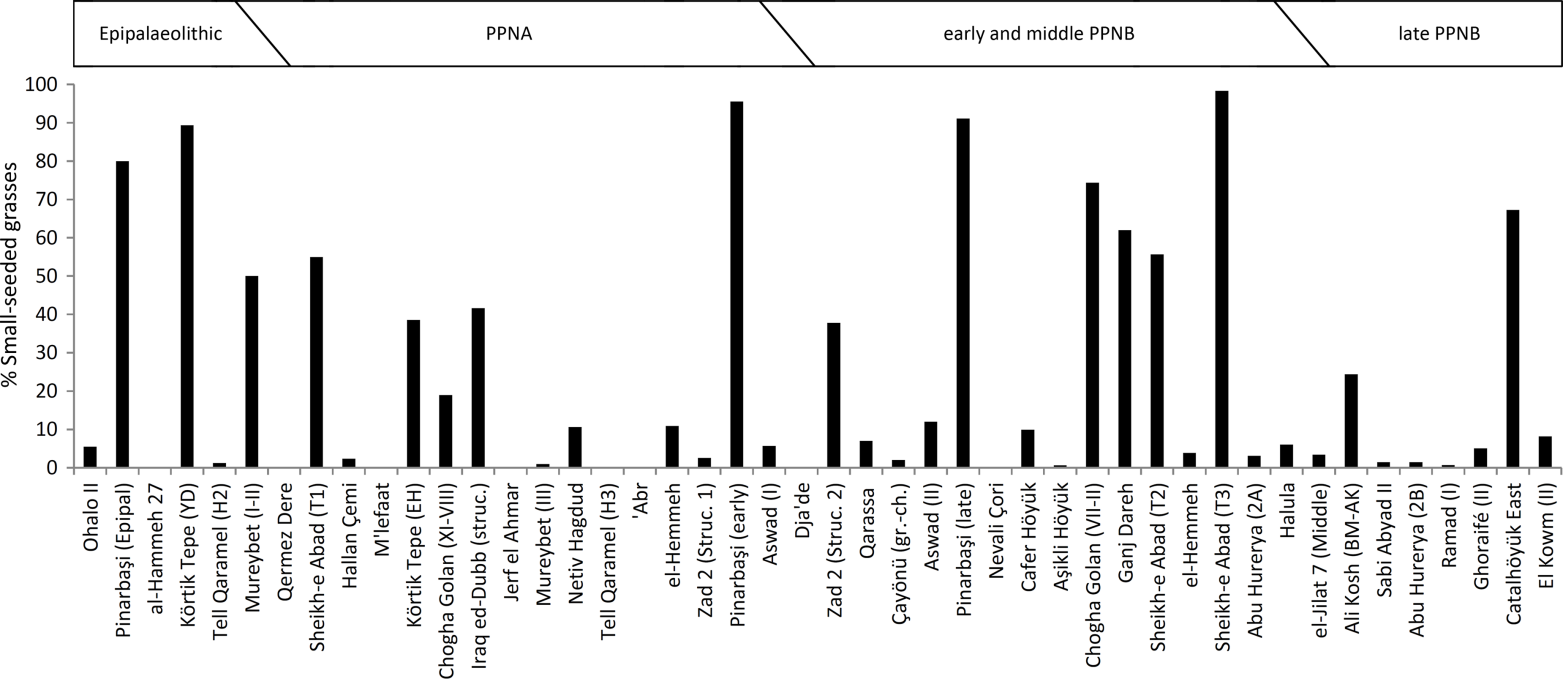
The proportions of small-seeded grasses among all Poaceae grains recovered from sites dating to the Epipalaeolithic and Pre-Pottery Neolithic. At sites that yielded > 60% of small-seeded grasses, types resembling *Agrostis*, *Alopecurus*, *Eragrostis*, *Phalaris*, *Phleum*, *Poa* or *Puccinellia* contributed the major portions.

Plotting the proportions of small caryopses through time did not reveal a temporal trend towards increasing or decreasing percentages (Fig 7). Instead, sites where small grasses clearly dominate the Poaceae grains are missing in the southern Levant. Here, the highest proportions of small caryopses occur at Epipalaeolithic Iraq ed-Dubb (exclusively *Phalaris*, 42%) and ZAD 2 (cf. *Poa bulbosa* bulbils and *Setaria* type, 38%). Colledge regarded the *Phalaris* grains as possible food sources or arable weeds [40], whereas Meadows did not explicitly discuss these small grains [33,72].

The highest proportions of small-seeded grasses with more than 60% of all grains occur, with increasing percentages, at Ganj Dareh, Çatalhöyük East (VI-PXII), Chogha Golan (AH VII-II), Körtik Tepe (Younger Dryas occupation), Pinarbaşi and Sheikh-e Abad (Trench 3). Most authors explained this phenomenon with the collection of herbivore dung or the grasses itself for fuel [18,39,57,90]. For Çatalhöyük, the combination of multivariate statistics, contextualized sampling and the application of common criteria to recognize dung-derived plant materials reliably demonstrated that dung was used as a fuel source throughout the whole occupation period [85,87,91]. As a result, small grains from taxa such as *Sporobolus*, *Aeluropus* or *Eragrostis* were abundantly brought into the site. Besides Çatalhöyük, such an approach to disentangle arable weeds, crops and dung-derived seeds was only applied to Sheikh-e Abad, where Whitlam also argued for dung burning as the most likely source for the high abundance of the Poa-type caryopses [18]. Riehl and her colleagues reject the dung-burning hypothesis for Chogha Golan and Körtik Tepe, because they view animal management as an important requirement for the routine use of dung as fuel and such evidence is absent at both sites [60,64].

#### The long-term development of selected wild grasses based on ubiquity values

A total of 47 sites/phases could be included in the analysis of wild grass ubiquity over time. Most identified Poaceae taxa in Epipalaeolithic and aceramic Neolithic assemblages occur infrequently among these sites and their identification rarely has major implications for reconstructing gathering or cultivation activities. In contrast, some genera are very frequent among the analyzed datasets, which possibly indicates a role in the prehistoric subsistence economies. Among these frequently identified non-cereal taxa, two different developments through time can be observed.

**Fig 8:**
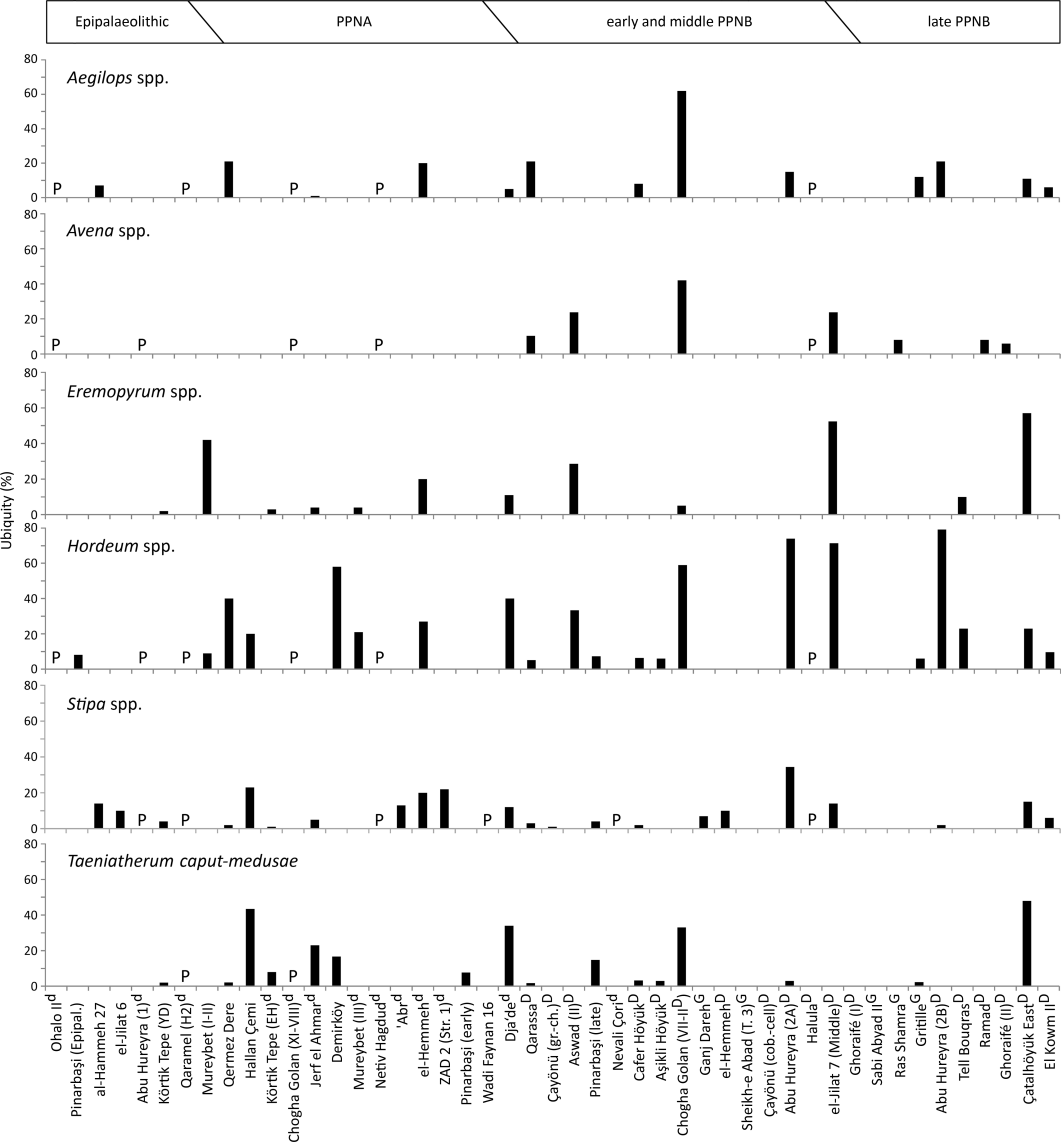
The ubiquity of grains from selected Poaceae taxa representing gathered resources at many Epipalaeolithic and PPNA sites and became common arable weeds during the PPNB. (P) present; (d) proposed pre-domestication cultivation; (D) presence of domestic cereal chaff; (G) domestic status of cereals based on grains.

The first set of taxa abundantly occurs at Epipalaeolithic to PPNA sites as well as during the PPNB (Fig 8). They do not follow an overall trend towards increasing or decreasing ubiquity values and show a relatively equal occurrence among the sites of the different periods. This set of taxa comprises species of *Aegilops*, *Avena*, *Eremopyrum*, *Hordeum*, *Stipa* and the monotypic genus *Taeniatherum*. Many authors consider them as gathered resources at Epipalaeolithic and PPNA sites, with food as the most frequently mentioned usage. Particularly remains of *Aegilops* spp., *Avena* spp., *Hordeum* spp., *Stipa* spp. and *Taeniatherum caput-medusae* seem to be uniformly interpreted in this way, given they occur with a certain abundance in the analyzed assemblages [28,51,60,73,74,88]. Other authors interpreted their occurrence more cautiously, mostly because they discussed pre-domestication cultivation for sites where wild cereals are clearly dominant [24,32,35,76]. Here we start to see a pattern where the relative abundance between wild cereals and other wild grass species seems to influence the interpretation of the wild grains as food sources or arable weeds indicative for cultivation. We should therefore be cautious to follow these original interpretations, if they are not supported by more detailed taphonomic analyses.

*Eremopyrum* species are relatively rare on most PPNA sites except for the early phases of Mureybet, where grains occur in many samples but always with low numbers. Van Zeist and Bakker-Heeres discussed a possible interpretation of *Eremopyrum* as an arable weed [24]. However, they note that the frequency of *Eremopyrum* grains and other potential weeds decreases alongside the increase of einkorn towards phase III. This seems to speak against an interpretation as crop-processing by-products and they leave the interpretation of these taxa undetermined.

With the appearance of domestic cereals during the early and middle PPNB, the abundance of these taxa does not substantially change. All above mentioned genera comprise species which occur as arable weeds in the Near East today [92,93]. Based to this, many archaeobotanists interpreted them as arable weeds at early and middle PPNB sites [23,40,77,79,81,94]. Others still considered these grasses as possible food resources at sites outside the Levantine corridor [18,39,44,57]. At Abu Hureyra and Wadi el-Jilat 7, where especially *Eremopyrum*, *Hordeum* and *Stipa* grains have very high ubiquities, de Moulins and Colledge did not rule out deliberate gathering activities [40,42]. These relatively diverse interpretations apparently reflect the difficulty to assess the route of entry of wild taxa into agricultural assemblages. Following this, for Cafer Höyük and Aşikli Höyük, these wild grasses were not interpreted [42,82].

The role of the considered grass taxa as arable weeds at late PPNB sites is mostly unquestioned [21,23,89,95,96]. For Çatalhöyük, the state of the wild grasses as arable weeds is supported by the results of the statistical analyses and the authors also discussed whether the *Taeniatherum caput-medusae* grain storages represent stored weeds outsorted from the crop harvests [41,53]. De Moulins had problems to solely interpret the abundant wild grasses at Abu Hureyra as weeds, where grass gathering seems to have had a long tradition [42].

**Fig 9:**
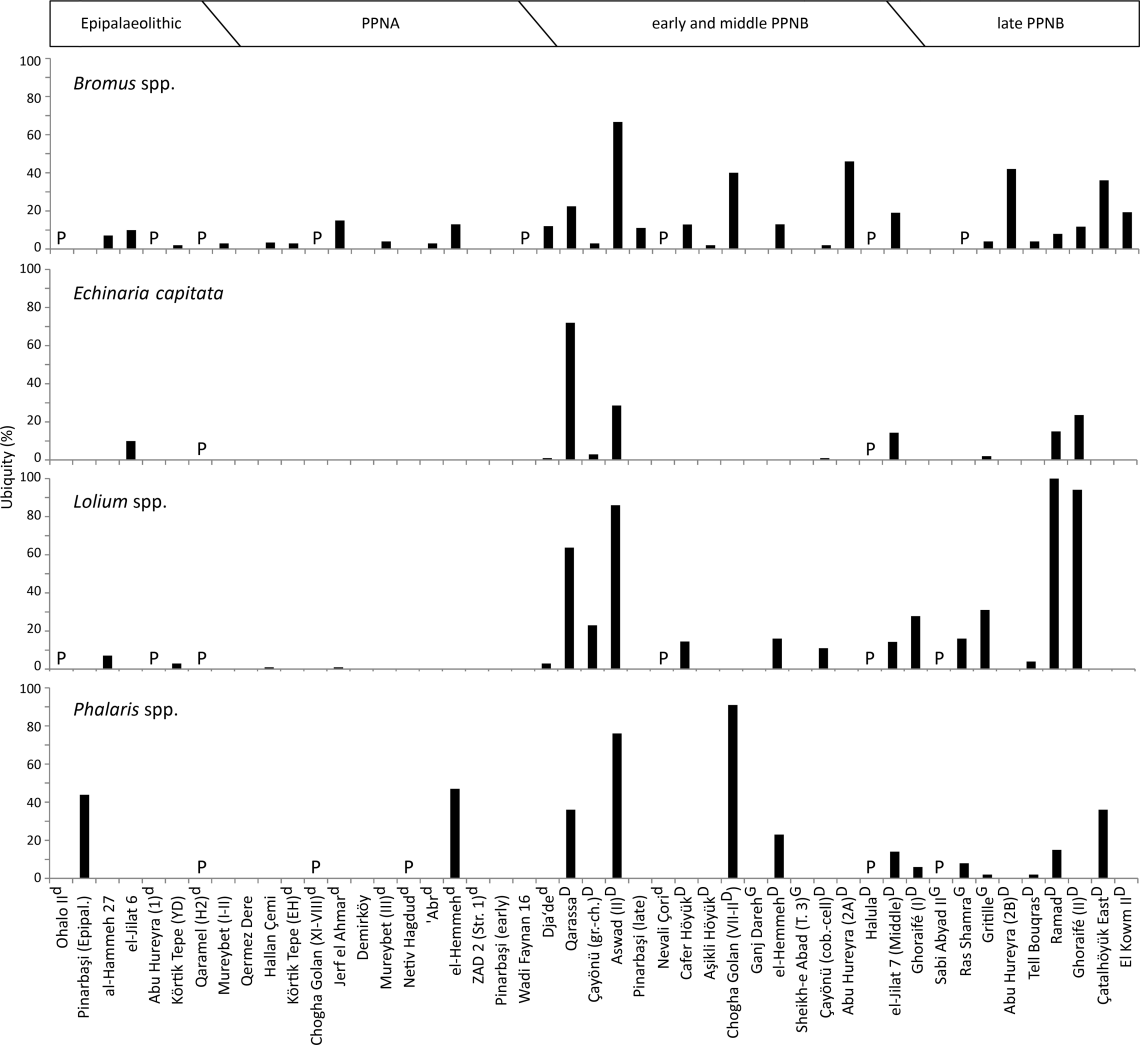
The ubiquity of grains from selected Poaceae taxa, which increase in abundance during the PPNB due to their establishment as typical arable weeds. (P) present; (d) proposed pre-domestication cultivation; (D) presence of domestic cereal chaff; (G) domestic status of cereals based on grains.

Fig 9 gives the development of ubiquity values for taxa that follow a different temporal pattern. *Echinaria capitata* and *Lolium* spp. grains substantially increase in abundance towards the PPNB, where they are present at much more sites than during the Epipalaeolitic and PPNA. *Bromus* spp. grains occur on most sites of all periods, but similarly show higher ubiquity values in the PPNB. The increase in abundance of *Phalaris* species can be seen in their frequency among the sites. *Phalaris* spp. grains are present at around 23% of the Epipalaeolitic to PPNA sites and at 52% of the PPNB sites.

Evidence for the exploitation of these taxa prior to the PPNB is limited. Weiss and his colleagues interpreted *Bromus pseudobrachystachys*/*tigridis* as gathered foods at Ohalo II, where the grains occur with high numbers in a plant-food preparation area in a brush hut [49,73,74]. *Lolium* sp. was presumably gathered at Sheikh-e Abad [18], but nowhere else and *Phalaris* may have been exploited at Pinarbaşi, Netiv Hagdud, el-Hemmeh and Chogha Golan (28,64,75,77). During the PPNB, all these taxa apparently became common arable weeds. *Lolium* species developed into the most successful segetals, whose grains are present in 94% to 100% of all samples from late PPNB Ghoraifé and Ramad [23]. A regional exception is Chogha Golan, where *Phalaris* and *Bromus* are abundant prior to the emergence of domestic emmer and may represent gathered resources.

## Discussion

### Subsistence strategies at Chogha Golan and in the Zagros Mountains

#### Explaining variability among the charred assemblages from Chogha Golan

We conducted a correspondence analysis to test, whether the analyzed flotation samples from occupation and midden deposits differ in their taxonomic composition. Interestingly, the samples followed a parabolic distribution in the scatter plot. Such a pattern is the result of a factor within the data table, which orders the samples along a specific gradient. This means that the samples at both ends of the ordered data table show the largest difference and all other samples are gradually more similar to one another. Such a pattern can be problematic, e.g. for ecologists, because the distances between the data points in a parabolic plot do not reflect the “real” distances between the observed cases. However, this issue is of limited importance in an archaeological application [97]. The cases that archaeologists use, for example artifact types that occur at sites in a given region, often follow a chronological order. Here, the absolute distances between the data points are of no interest, because the aim is to order the sites according to their relative chronology [98].

At Chogha Golan the cases represent flotation samples characterized by their taxonomic composition, which derive from the deep sounding of a tell excavation. The parabolic distribution of the samples therefore means that their composition follows a strong temporal trend throughout the sequence, which allows us to interpret the compositional differences between the samples as a temporal development. The type of deposit is only a secondary factor influencing the formation of the charred assemblages. As an exception to this overall pattern, only three samples from the midden deposits of AH VI are clearly separated from the other samples along axis one, because possibly gathered seeds of *Atriplex* sp. and *Alyssum* sp. and bulbils of *Poa bulbosa* accumulated here.

#### Wild grasses in the subsistence of Chogha Golan

In the first archaeobotanical reports about Chogha Golan the presence of cereals, their status as pre-domestic or domestic cultivars and the high proportions of small-seeded grasses between AH V and III were discussed in detail [16,27,44,56,64]. Therefore, the significance of the new results lies in the high amount and diversity of the large to medium-seeded wild grasses. Their grains are considerably more abundant among the analyzed samples from horizons VII to III than grains of *Hordeum spontaneum*, *Triticum* spp. and *Aegilops* sp., which also applies to the older horizons XI to VIII [16,64]. These high proportions of large to medium-sized grains from wild *Avena*, *Bromus*, *Eremopyrum* and *Hordeum* species, the indeterminate Triticoid type and *Taeniatherum caput-medusae* raise the question of whether they were exploited as food resources and how their state in the subsistence economy relates to the wild cereals.

We previously argued that *H*. *spontaneum* and possibly even *Aegilops* sp. were cultivated at Chogha Golan [16,44,64]. Such pre-domestication cultivation systems are traditionally seen as scenarios in which grains of wild species are sown on tilled soils where they experience enhanced growing conditions and are accompanied by arable weeds [6,15,16,28,31,32,35,37,99]. Applied to Chogha Golan this scenario could explain the high abundance of wild cereal remains, the temporal grain size increases of *H*. *spontaneum* and the presence of many wild grasses representing potential segetals. However, in light of accumulating archaeobotanical data for Chogha Golan and other aceramic Neolithic sites in the Zagros Mountains, we became increasingly cautious in interpreting the observed patterns using this explanatory framework. Although the patterns from Chogha Golan basically fulfill the criteria commonly used to apply the pre-domestication cultivation hypothesis, we see considerable differences between this dataset and those from the Levantine corridor.

Central for the proposition of pre-domestication cultivation, particularly for sites in the upper Euphrates area, was the interpretation of several potential weeds as representative of an early segetal flora, but also the gradual and clear decrease of gathered resources contemporaneous to a marked increase in wild cereals [32,36]. This development resulted in a strong dominance of wild cereal remains and only minor portions for other wild grasses at all PPNA sites of this region (see discussion below). Contrastingly, the sequence at Chogha Golan does not display a major shift towards an increasing focus on wild cereals and a gradual decrease of gathered wild plants. These data seem to indicate constant exploitation strategies throughout the sequence and are in good accordance with datasets from other sites in the Zagros Mountains (see discussion below and [18,51,56]). In fact, the patterns seem to be reversed here, with diverse large to medium-seeded grasses dominating the assemblages and wild cereals only representing a few among many important food resources.

Applying the traditional concept of pre-domestication cultivation to Chogha Golan would ignore all these patterns, which we identified during our ongoing analyses. In light of this conclusion we have to ask how we will now characterize this subsistence economy? We are left with the finding that *H*. *spontaneum* and *Aegilops* sp. presumably represent staple foods and that they were routinely exploited throughout the entire occupation period. Large to medium-seeded grasses exhibit the same patterns and, following the general picture for wild grass exploitation in the Zagros Mountains, they presumably represent routinely gathered foods as well. However, we are still confronted with the exploitation of diverse wild grasses throughout an occupation period of about 2,000 years. During the major part of this period, but at least 1,000 years, the inhabitants constructed permanent architecture indicative of a sedentary community. This leads us to the question if humans could continuously rely on these resources without any form of resource management? A look at the taxonomic composition of the identified wild grasses with regard to their present day ecology might give an additional clue for reconstructing subsistence practices.

**Fig 10:**
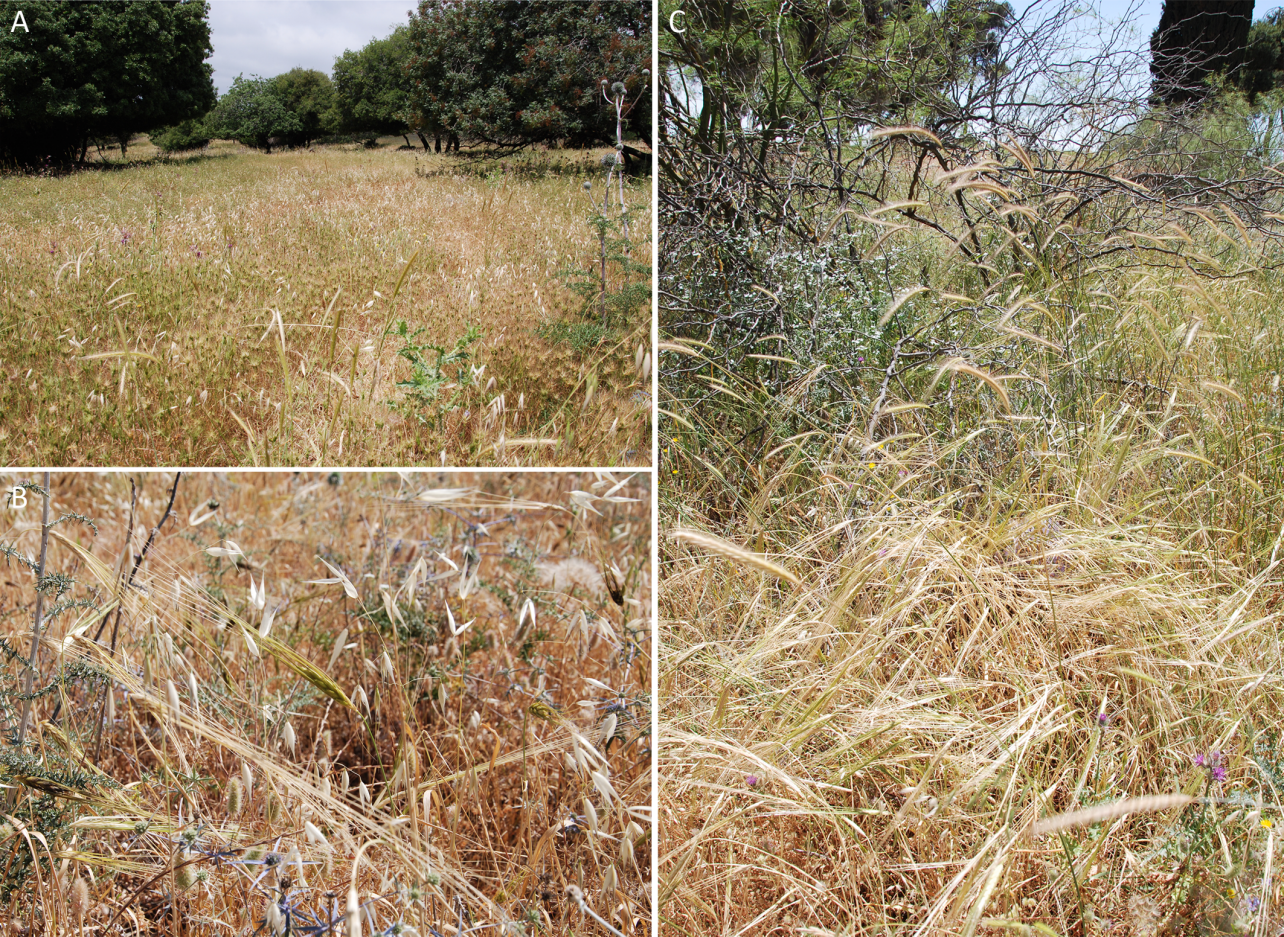
Associations of wild cereals and other wild grasses in northern Israel. (A) Rich stands of *Aegilops peregrina* in open woodlands of *Quercus calliprinos* and *Pistacia palaestina* at Mount Carmel, accompanied by *Hordeum bulbosum*, *Bromus lanceolatus* and *Avena sterilis*, whereas *Hordeum spontaneum* represents a minor component; (B) ruderal habitat along the road between Rosh Pina and Safed in the Upper Galilee Mountains, dominated by *Avena sterilis* and *Aegilops peregrina* and accompanied by *Hordeum spontaneum* and *Triticum dicoccoides*. Wild emmer wheat even grew within the village of Safed on waste places, which it apparently invaded from nearby primary stands; (C) same location near Safed, only about 50m away; dominated by *Hordeum spontaneum* (ears below) and *Hordeum bulbosum* (ears above) with *Triticum dicoccoides*, *Avena sterilis* and *Aegilops peregrina* as minor components. These wild stands display a considerable diversity in taxonomic composition on a small spatial scale. Small to medium-seeded taxa such as *Bromus* spp., *Lolium rigidum*, *Poa bulbosa* and *Phalaris* spp. were regularly associated with these wild cereal stands. Photos by A. Weide, 2017.

In extant Near Eastern ecosystems, where we find the species under discussion, wild cereals, including *Aegilops* spp., often dominate the herbaceous vegetation and grow together with e.g. *Avena sterilis*, *Bromus* spp., *Hordeum bulbosum* or *Taeniatherum caput-medusae* (Fig 10) [66,93]. It is important to note that many of these taxa include well known synanthropic species, today adapted to ruderal and segetal habitats, which substantially affects their present day distribution [92,100,101]. Using the modern ecology of wild cereals and other wild grasses for interpreting prehistoric botanical assemblages therefore includes the danger of drawing false assumptions based on differing ecological adaptations in present and ancient ecosystems. However, the botanical remains from Chogha Golan are surprisingly similar in their taxonomic composition to these extant stands, suggesting that the exploited early Holocene grasslands were not very different from populations we can observe today. This finding possibly indicates that the wild cereals and grasses at Chogha Golan were gathered from “natural” stands rather than harvested from a cultivated field. Moreover, the fact that most wild cereals and many of these wild grasses developed into successful synanthropic species and dominate many primary as well as secondary habitats today raises the question of how early these traits developed and if they played a role in the ability of the inhabitants of Chogha Golan to exploit them as major food resources for almost two millennia? We will not be able to draw final conclusions on this issue in the present paper, also because more research in this field is needed, but we want to emphasize the importance of the potential synanthropic ecology of many of the wild grasses under discussion. As we will demonstrate below, several typical segetals abundantly occur among charred botanical assemblages as soon as domesticated cereals appeared in the early PPNB. These taxa were able to quickly adapt to human disturbances, in this case soil tillage and crop cultivation. We can therefore principally expect that some extant synanthropic species could immediately adapt to anthropogenic disturbances by prehistoric humans [38] and, as a result, were possibly “pulled” into the sedentary human environment.

In the surroundings of Chogha Golan, the exploited wild grasses were presumably favored by anthropogenic disturbances, which inevitably and increasingly must have occurred after humans became sedentary. The creation of disturbed and open habitats did not play a primary role in the choice of the inhabitants to exploit wild grasses as major foods, because local palaeoenvironmental data indicate generally open landscapes with a large grass component during the early Holocene [102,103]. However, they could have facilitated the long-term exploitation of wild grasses. Size increases of *H*. *spontaneum* grains indicative of enhanced growing conditions towards AH IX and IV possibly point towards deliberate management practices [64], but their exact nature is yet to be established.

Independent from these issues we can conclude that the inhabitants of Chogha Golan developed an effective and sustainable subsistence strategy, which allowed a sedentary lifestyle for at least one millenium, if not substantially longer, without relying on domesticated plant resources. This subsistence strategy must be characterized as a resilient long-term solution and not as “on the way” to domestication, which the term pre-domestication cultivation would imply. The only obvious and presumably permanent shift in this subsistence economy was the adoption of domesticated emmer wheat towards the end of occupation at the site. This coincided with decreasing percentages of the formerly exploited wild grasses, representing an additional argument for why we consider the wild cereals and the large to medium-seeded grasses as representative of one uniform exploitation strategy. Otherwise they would not have equally competed with domesticated emmer wheat.

### Patterns in subsistence economies of the Zagros Mountains

The archaeobotanical remains from Chogha Golan with the high proportions of non-cereal taxa are in good accordance with results from many other broadly contemporaneous sites of the eastern wing of the Fertile Crescent. These include East Chia Sabz and Sheikh-e Abad in the central Zagros [18,56] as well as M’lefaat and Qermez Dere, located in the Mesopotamian plain in northern Iraq (51). At all these sites wild grasses, including species of *Hordeum*, *Lolium*, *Piptatherum*, *Stipa*, *Taeniatherum*, and the Triticoid type, are abundant and accompany the cereals. Savard et al. and Whitlam clearly regard these wild taxa as gathered food resources [18,51]. At East Chia Sabz and M’lefaat, *Aegilops* grains or chaff outnumber those of *Hordeum spontaneum*, supporting our view that one or several *Aegilops* species were among the local staples, whose role in the subsistence economies was equally important than that of *H*. *spontaneum* [44]. Towards the Taurus Mountains, patterns in subsistence strategies seem to have been different. The wild grasses at Körtik Tepe, predominantly small-seeded taxa, are regarded as staple foods that decrease in importance with the onset of the early Holocene [60]. This development matches the data from Hallan Çemi and Demirköy, where Savard et al. found valley-bottom species of *Rumex*, *Polygonum* and *Bolboschoenus* to be the major food plants [51]. The high importance of large to medium-seeded wild grasses in the diet of early Holocene sedentary communities therefore seems to be a regional pattern in the Zagros Mountains and the adjacent Mesopotamian plain, where domestic plants appear later than in the Levantine corridor [19].

Wild grasses start to decrease in abundance in the central Zagros with the onset of the 10^th^ millennium cal. BP. At Chogha Golan, this correlates with the emergence of domestic emmer in AH II at around 9,800 cal. BP. These finds of domestic emmer chaff currently represent the oldest evidence for a non-shattering cultivar in the eastern wing of the Fertile Crescent and seem to mark a major change in regional subsistence strategies. The assemblage from trench 2 of Sheikh-e Abad still contains a considerable portion of gathered large to medium-seeded grasses and is roughly of the same age as the middle and upper horizons of Chogha Golan [18]. Domestic-type barley grains have been identified, but the subsequent development is hardly detectable, because the samples from the youngest layers in trench 3 are heavily dominated by small-seeded grasses. Whether the proposed onset of barley cultivation at Sheikh-e Abad resulted in a decreased importance of other wild grass resources can unfortunately not be evaluated due to this huge taphonomic bias.

All sites and settlement phases in the central Zagros, which roughly date to or are younger than 9,800 cal. BP, show a different pattern. At Ganj Dareh, van Zeist et al. found a minor component of large to medium-seeded grasses accompanying domestic-type barley grains [39]. Whether these grains derive from non-shattering barley ears is impossible to assess, but the overall patterns continue a trend where wild grasses show decreasing proportions. At the subsequent sites of Ali Kosh and Chogha Bonut [21,104], the few wild grasses most likely represent weeds and emmer, barley and lentils are well established as domestic crops throughout the central Zagros by the middle of the 10^th^ millennium cal. BP [90].

### The role of wild grasses at hunter-gatherer and farming sites of the Fertile Crescent

#### Epipalaeolithic and PPNA: Gathered resources compete with early cultivars

Almost all Epipalaeolithic sites with a rich record of macro-botanical plant remains provide evidence for wild grass gathering and their state as important food resources. Weiss et al. suggested that wild grasses were part of the food diversification during the late Pleistocene [48], which Flannery originally named the “broad spectrum revolution” [50]. Among the routinely exploited grasses of the Epipalaeolithic, small and medium-seeded taxa such as *Alopecurus*, *Bromus* or *Hordeum* similarly contributed to the grain harvests as opposed to the wild cereals. However, this pattern is based on a limited number of sites and only phytolith analyses from the two Late Natufian sites of Eynan and Hilazon Tahtit in the Hula valley and Galilee provide additional evidence for the frequent exploitation of wild grasses and cereals [105]. We therefore have to speculate whether wild grasses were continuously gathered throughout the Epipalaeolithic and the phytolith evidence rather points to shifting subsistence strategies, depending on the changing availability of plant resources through space and time. Phytolith evidence from Kharaneh IV in the Azraq basin in Jordan, combined with Ohalo II, indeed suggests that localized year-round habitations during the Early-Middle Epipaleolithic in the southern Levant were mainly based on wetland resources and not primarily on the exploitation of wild grasses and cereals [58,106,107].

Even though Snir et al. saw the earliest evidence for wild cereal cultivation at Ohalo II [38], it is not until the end of the Epipalaeolithic and the onset of the early Holocene in the Levantine corridor that the wild progenitors were primarily used as staple foods. During the early phases at Qaramel, Mureybet and Abu Hureyra, wild cereals were increasingly exploited, indicating the beginnings of a major change in human subsistence strategies. All sites in the Levantine corridor dating to the PPNA now show a very pronounced focus on wild cereals, which most scholars explained with the emergence of wild cereal cultivation (28,32,35,40,48,52).

Although non-cereal taxa occur on many of these sites, sometimes in considerable quantities, and many authors regard these grains as potential food sources, wild grass gathering has been regarded as negligible in importance after the onset of the early Holocene. Weiss and his colleagues came to this conclusion by examining a selection of Levantine sites and calculating the ratio of the cereals compared to the other wild grasses with respect to the steep gradient of declining grain volumes. It is absolutely right that the non-cereal taxa did not contribute substantial proportions to the whole grain harvests after humans began to focus on cereals. However, studies accomplished during the last ten years provided evidence for the possible exploitation of wild grasses during the PPNA in the Levantine corridor, even though most of them focused on the interpretation of the wild cereals [32,35,52,76,78,108]. We must therefore adjust our presumptions about the demise of wild grass exploitation with the onset of the early Holocene.

A consistent line of evidence we found for the PPNA in the Levantine corridor indicates a continuous tradition of wild grass use at many sites, although wild cereals start to heavily dominate the assemblages. The accumulated finds and high counts for wild grasses such as *Aegilops*, *Avena*, *Hordeum*, *Stipa* or *Taeniatherum* at sites such as Netiv Hagdud, el-Hemmeh, Qaramel, Jerf el Ahmar or Dja’de could theoretically be explained by their state as arable weeds in pre-domestic cereal fields, but there is no contextual evidence at either of these sites that would support this conclusion. Typical segetals only increased in abundance with the appearance of domestic cereals in the early and middle PPNB, but all above mentioned taxa were already ubiquitous throughout the Epipalaeolithic and PPNA. This is at best explained by their continuous exploitation as food resources, most apparent at Gilgal, where *Avena sterilis* and *Hordeum spontaneum* grains were found together in one storage context [52,78]. Weiss et al. were right when they stated that the cereals replaced the wild grasses as major foods, but new data indicate a continuous exploitation of wild grasses by PPNA communities in the Levant, which were only later fully replaced by domestic cereals in established agricultural systems. However, we must keep in mind that these interpretations are mostly based on the relative frequency of wild grass and cereal remains. As we will more clearly point out below, comprehensive taphonomic analyses using a multivariate approach should be applied to datasets suited for such an analysis in order to more reliably disentangle arable weeds and deliberately gathered resources.

Another issue is the study region. The origins of agriculture are traditionally seen in the Levantine corridor [109–111]. Hence, many studies on the subsistence development in the early Holocene focused on this region, although recent evidence suggests multiple trajectories towards farming in different regions within the Fertile Crescent [15,16]. In this paper we present additional evidence for a significant regional variability in subsistence strategies, supporting previous evidence for a reliance on wild grasses in the Zagros Mountains during the 12^th^ and 11^th^ millennia BP [18,51]. Here, in contrast to the Levantine corridor, wild grasses were not replaced by the wild cereals as major food sources after the end of the Younger Dryas. Although different climatic and environmental conditions contributed to an uneven availability of wild cereal species throughout the Fertile Crescent [9,59,112], they were present and have been exploited in the Taurus-Zagros Mountains from the very beginnings of the Holocene onwards [18,51,60,64]. *Triticum* species are almost absent from this record, but *Aegilops* is present in high quantities at M’lefaat, Chia Sabz and Chogha Golan and occurs frequently among the samples from Qermez Dere. This again highlights why we include *Aegilops* in the group of wild cereals in the Zagros Mountains, which possibly compensated for the lack of wild *Triticum* stands.

Taking the Fertile Crescent as a whole, *H*. *spontaneum* was routinely exploited in all regions and has been accompanied by *T*. *dicoccoides* and *Avena* spp. in the southern Levant, one and two-seeded einkorn and rye in the northern Levant and *Aegilops* spp. in the Zagros Mountains. As Fuller et al. already pointed out, this extended list of intensively exploited and increasingly managed wild cereals characterized subsistence economies during the PPNA, although not all of these species became established as a domestic crop during the following periods [15]. Based on this evidence, the absence of a heavy focus on wild cereals in the Zagros cannot be explained by a lack of wild cereal stands. Alternatively, the ecosystems in the eastern wing of the Fertile Crescent seem to have been exploited in a different way that was equally successful. Discussing possible reasons for such different trajectories is beyond the focus of this paper, but we want to refer to important contributions that discuss early cultivation in the light of evolving sedentism, monumental architecture, food storage, feasting, symbolism and emerging property, which are highly entangled with one another in the archaeological record, suggesting the socio-cultural background as a determining factor influencing the development of subsistence strategies [113–120].

#### PPNB: Invading the fields

With the appearance of domestic cereals in the early and middle PPNB, many grasses became common arable weeds. We could identify two different patterns that are indicative of this development. A first one in which formerly exploited grasses seem to have invaded fields and are equally abundant at sites occupied by hunter-gatherers and farmers. These taxa include species of *Aegilops*, *Avena*, *Eremopyrum*, *Hordeum*, *Stipa* and *Taeniatherum*, all of which include known segetals today and have been interpreted as such by the respective authors at PPNB sites. The second pattern is representative for species which considerably increase in abundance with the appearance of domestic cereals, suggesting that they are more indicative of arable weeds in the overall archaeobotanical record than as gathered resources. Among them, *Lolium* spp., *Phalaris* spp. and *Echinaria capitata* show the most profound increase in abundance and occur on more sites and in greater abundance during the PPNB. However, these taxa are not fully absent from the list of potentially gathered resources in the earlier periods. Particularly *Bromus* is present on most Epipalaeolithic and PPNA sites, but then occurs more frequently in the charred assemblages of farming villages. Likewise, *Phalaris* species seem to have been exploited at some PPNA sites, possibly also at Chogha Golan, but then clearly developed into successful arable weeds. An interesting pattern is the increasing abundance of *Echinaria capitata* in the southern Levant and at Halula at the upper Euphrates [108]. The species is not known as a typical arable weed today [92,93], but was apparently part of PPNB segetal communities.

The overall proportions of large to medium-seeded wild grasses do not substantially decrease after the PPNA. Particularly at late PPNB sites such as Sabi Abyad II, El Kowm and Ghoraifé, they outnumber the cereal grains. Apparently, *Lolium* species have developed into successful segetals, making up the majority of arable weed remains at these sites. Here it is important to note that the weeds are not solely representative of cereal agriculture. Van Zeist examined the proportions of *Lolium* grains for sites in the Balikh basin in northern Syria, which show a fair correlation with *Linum* sp. seeds, indicating that *Lolium* species were major weeds in flax fields [89]. The continuously high percentages of wild grasses also highlight the different taphonomic agents that now contributed to the formation of charred archaeobotanical assemblages. As de Moulins discussed [42], wild plant use is hardly detectable as soon as farming developed, because crop-processing by-products will end up in the charred plant materials. Without applying multivariate methods it is then often impossible to identify the deliberately gathered species. This is a well-known problem and has been addressed by many studies, who sought to disentangle the archaeobotanical assemblages in this regard [18,24,37,40,43,44,81,87,91]. Concerning the wild grasses that have been exploited during the Epipalaeolithic and PPNA, but still frequently occur in PPNB assemblages, we are mostly left with the overall assumption that these taxa now represent weeds and not gathered resources. A central argument for this interpretation is that the cultivation of domestic cereals makes wild grass gathering redundant, because they would not significantly contribute to the grain diet [48,53]. Moreover, the substantial increase of major segetal taxa like *Bromus* or *Lolium* since the early PPNB is indicative of developing crop-processing techniques, which increasingly contribute to the formation of charred botanical assemblages at PPNB sites including high proportions of weed seeds.

Despite this general agreement, several authors were confronted with patterns or features that led them to discuss a potential utilization of wild grasses at middle and late PPNB sites such as Abu Hureyra, Wadi el-Jilat 7 or Çatalhöyük [40,42,53]. For Abu Hureyra, de Moulins emphasized the taxonomic composition of the PPNB plant remains, which is strikingly similar to the Epipalaeolithic remains of the site. At most middle or late PPNB sites where wild grasses make up large proportions of the plant remains they are commonly dominated by a few weeds such as *Bromus* sp. or *Lolium* sp. In contrast, the abundant wild grasses at Abu Hureyra include many more taxa, of which *Stipa*, *Secale* or wild *Hordeum* species were interpreted as exploited resources during the Epipalaeolithic occupation [121,122]. This pattern may indicate that most taxa gathered during the Epipalaeolithic entered cultivated fields as arable weeds during the PPNB at the site, or it is indicative of a continuous exploitation of these grasses. The information we currently have do unfortunately not allow to distinguish between these two hypotheses, emphasizing the need for more detailed taphonomic analyses.

At Wadi el-Jilat 7, it is the location in the “marginal zone” of the environments in the southern Levant that provides an argument to have a closer look at the wild grasses. The development of distinct subsistence strategies has long been recognized for the drier and steppic regions of the southern Levant, where archaeological sites have a more ephemeral character in contrast to the Mediterranean zone with its rich record of sedentary farming villages [123,124]. Although Garrard and colleagues solely discussed the finds of domestic crops for sites in the Azraq basin in eastern Jordan [54], Colledge included the abundant non-cereal taxa dominated by *Eremopyrum* and *Hordeum* into her list of plants that “would have provided sources of food” at Jilat 7 [125]. As indicated by this dataset, wild grasses were possibly exploited alongside domestic cereals in environments bordering the deserts of the Sinai, Negev and Jordan, from which informative archaeobotanical datasets are almost absent. Although this must remain hypothetical, we should be cautious in regarding wild grasses as generally outcompeted and unattractive resources among PPNB societies, which apparently display a considerable diversity in settlement patterns and subsistence strategies and of which some continued to live a more mobile lifestyle in these “marginal environments” [55,126]. Furthermore, it is conspicuous that wild *Hordeum* and *Eremopyrum* grains, but also *Avena* and *Stipa*, reach their most frequent occurrence during the PPNB at Abu Hureyra, Jilat 7, Chogha Golan and Çatalhöyük (see Fig 8), which are all sites where wild grass exploitation has been addressed.

At Çatalhöyük this was inferred by the two possible storage finds of *Taeniatherum caput-medusae*, accompanied by *Eremopyrum*-type grains. Both taxa seem to represent common weeds at the site [41,53] and this particular case highlights the importance of singular contexts for our understanding of wild plant exploitation in farming societies. Although Fairbairn and his colleagues are cautious in interpreting *Taeniatherum caput-medusae* as a stored food, its accumulated appearance in two subsequent settlement phases clearly speaks for a planned utilization of the grains. Apparently, the status of a taxon as a common arable weed, like *Taeniatherum caput-medusae* in Anatolia, cannot be taken as a strict indicator of its role in subsistence economies. Modern societies have very different conceptions of weeds [127,128] and Hillman reported an interesting case of Anatolian families preferring a bread from wheat harvests that were “severely infested” by weedy forms of *Secale cereale* and *Vicia sativa* [129]. In contrast to the concept of a weed as an undesired plant without any value, early Holocene people could have accepted, utilized and probably even supported edible weed species, particularly as many segetals represented exploited resources prior to the emergence of crop cultivation. The patterns from Abu Hureyra and Çatalhöyük may be indicative of such practices, which White also discussed for el-Hemmeh [80]. By specifically addressing this issue in future archaeobotanical analyses, implementing multivariate methods more intensively, we can hopefully shed more light on the treatment and status of arable weeds in prehistoric subsistence economies.

#### The small-seeded grasses

Accumulations of small caryopses heavily dominate some charred archaeobotanical assemblages we considered in the present study. This phenomenon occurs from the Epipalaeolithic through to the late PPNB, mainly in the Zagros Mountains and Anatolia. Types resembling *Agrostis*, *Alopecurus*, *Eragrostis*, *Phleum*, *Poa* and *Puccinellia* have been reported as the dominating taxa contributing to these assemblages. The classification of nearly all taxa as “cf.” or “types” illustrates the difficulty of identifying these charred grains, which are extremely small and are often measure less than 1mm in length. Recovering large amounts of such small-seeded grasses require particular recovery techniques. We are therefore confronted with the question to which degree methodological aspects, such as the mesh sizes applied during water flotation, bias the observed patterns.

By plotting the proportions of small grasses among all Poaceae grains against the applied mesh sizes, we found that sieving seems to have an impact on the abundance of small caryopses, given the mesh sizes were 0.3mm or larger. However, we do not know whether the lower percentages of small grains at sites where larger meshes have been used are really due to a methodological bias. Willcox et al. tested mesh sizes smaller than 0.5mm at Jerf el Ahmar, Dja’de, Qaramel and ‘Abr without retrieving significant amounts of identifiable plant remains (they did not indicate which mesh sizes were tested) [32]. In addition, at many sites where mesh sizes smaller than 0.3mm have been used, small caryopses are likewise rare or absent. Field methods, as far as this can be evaluated using the published literature, therefore seem not to be a major cause of variation for the proportions of small-seeded grasses, which Weiss and colleagues similarly concluded [48].

When we assume taphonomic processes as the main factors causing this variability, we are confronted with a geographical rather than a temporal pattern. As concluded above, the Taurus-Zagros Mountains and central Anatolia display considerably different developments in subsistence practices as opposed to sites in the Levantine corridor. All sites with small-seeded grasses making up more than 60% of the Poaceae grains are located here, which suggests local subsistence practices being a major factor that induces this pattern. In the Zagros Mountains, subsistence strategies are characterized by the exploitation of wild grasses for food and the small-seeded taxa possibly represent deliberately collected resources as well. Riehl and her colleagues favor this explanation for Chogha Golan and Körtik Tepe, whereas the collection of grasses or herbivore dung as fuel has been suggested by most other authors [18,39,57,90].

Fairbairn et al. reported dung-pellets for the early phases at Çatalhöyük and considered particularly *Bolboschoenus* nutlets as being dung-derived, because their ecology made sedges the most plausible taxa being grazed by livestock and not harvested with the grown crops [41]. Further studies could later confirm that the *Bolboschoenus* nutlets as well as the small grains from *Aeluropus* sp., *Crypsis* sp., *Eragrostis* sp. and *Sporobolus* sp. are indeed dung-derived [85,87,91], whereas the origin of the abundant cf. *Alopecurus* grains from the early levels remains unclear. Çatalhöyük is therefore the only early Neolithic site where most small-seeded grasses could reliably be linked to dung-burning. The comprehensive application of multivariate statistics, in combination with a good situation for contextualized sampling and the application of established criteria to recognize dung-derived plant materials, represents the basis for these very informative archaeobotanical studies. Such a comprehensive approach was/could not be applied the other Anatolian or Zagrosian sites and we are therefore left with a quite unsatisfactory picture, which does not allow us to draw robust conclusions on the high proportions of small-seeded grasses at the sites under discussion. We should not take abundant small-seeded grasses *per se* as indicators for dung-burning, because different taphonomic factors also resulted in the incorporation of large to medium-sized grains in charred assemblages and include gathering and crop-processing. Whitlam eventually noted that herbivore dung as a major source for large amounts of small-seeded grasses in charred assemblages must remain hypothetical unless dung pellets including identifiable grains are found [18] and we fully agree with this assumption. However, it needs to be emphasized that a multivariate approach is currently the most promising way to reconstruct the taphonomic history of burned plant remains and must be applied more regularly in the future to proceed with solving such interpretative problems.

## Synthesis, main conclusions, and a future perspective

Chogha Golan provides an exceptional case for analyzing the development of early Neolithic subsistence strategies and for evaluating the importance of gathered resources in relation to emerging crop cultivation. The inhabitants of the Neolithic village routinely exploited a complex of wild grasses comprising wild cereals, large to medium-seeded and possibly also small-seeded taxa. *Hordeum spontaneum* formed a major component of the consumed grasses and was harvested for almost 2,000 years without inducing the establishment of non-shattering ears. Then, as soon as domesticated emmer wheat dominates the cereal remains by ca. 9,800 cal. BP, all wild grasses decrease in relative abundance. This complex of wild grasses was presumably harvested as staple foods and was subsequently outcompeted by a domestic crop. Whether the wild cereals were ever cultivated at Chogha Golan is therefore not clear and we consider the ability of the exploited wild grasses to invade anthropogenically disturbed habitats around the sedentary village as an important factor that possibly facilitated their sustainable long-term exploitation. To which degree management practices were applied to maintain the wild grass stands is unclear, but this scenario significantly deviates from the traditional concept of pre-domestication cultivation and must be seen as a successful and resilient long-term subsistence strategy that did not heavily focus on wild progenitor species.

The archaeobotanical results from Chogha Golan are in good accordance with the overall patterns of the aceramic Neolithic in the Zagros Mountains. Wild grasses were exploited as major food sources at many sites in the uplands and in the foothills bordering the Mesopotamian plain throughout the 12^th^ and 11^th^ millennium cal. BP. This is in sharp contrast to the development in the Levantine corridor, where wild grasses formed important components of Epipalaeolithic subsistence strategies and were gradually replaced by wild cereals as major food sources since the end of the Younger Dryas.

For evaluating wild grass gathering in relation to the emergence of cereal cultivation, we analyzed the occurrence of wild grasses throughout almost 15,000 years considering the entire Fertile Crescent. We were able to visualize spatial patterns and long-term developments, which we regard as crucial for interpreting the role of wild grasses throughout the aceramic Neolithic. We provide additional evidence for a considerable diversity among early Neolithic subsistence strategies on the inter-regional level and conclude that many wild grasses were gathered for food, fuel or other purposes from the Epipalaeolithic onwards and demonstrably into the PPNA period. Many of these gathered grasses were part of the earliest segetal communities that emerged with the beginnings of farming, emphasizing the potential dietary value of these weed species. This pattern is of major importance for evaluating the status of weeds in early farming communities. The archaeobotanical datasets from e.g. Çatalhöyük, Abu Hureyra or el-Hemmeh suggest the utilization of arable weed species, which is plausible in light of this result. A high diversity of wild grasses also co-occurs with domestic cereals at some sites in the “marginal zones” of the Levant, such as Jilat 7, which presumably indicates the utilization of these species by groups that maintained a more mobile lifestyle.

Finally we want to emphasize the great potential multivariate approaches have to reconstruct prehistoric subsistence practices. Our review unmistakably shows that we will not proceed in disentangling arable weeds, deliberately collected food resources and dung burning activities if we do not implement the full spectrum of analytical techniques available to date. Among these, multivariate statistical methods such as correspondence analysis, coupled with contextualized sampling from relatively extensive excavations, represent the most promising approach that has been successfully applied to a small number of sites. It is clear that some assemblages are not suited for such analyses due to a poor preservation of the plant remains or small excavation areas, but many published assemblages fulfill these criteria and can be investigated in future analyses to more reliably interpret the role of wild resources in relation to emerging cultivation and domestication.

## Supporting information

S1 Table Average 1000 seed weight of Poaceae species. Based on the Seed Information Database of the Royal Botanic Gardens Kew [67].
(DOCX)

S2 Table Analyzed flotation samples and identified plant remains from AH II to VII from the deep sounding of Chogha Golan. (DOCX)

S3 Table Coding of the variables in the correspondence analysis. (DOCX)

S1 Fig The site of Chogha Golan. (A) The landscape around Chogha Golan; (B) outline of the site with the location of the deep sounding and excavation Area A in the center of the tell; (C) the south profile of the deep sounding showing Archaeological Horizons I to XI and the related z-values. Figures and photos by M. Zeidi.
(TIF)

S2 Fig Average grain weight of Poaceae taxa frequently identified among charred archaeobotanical assemblages from the Near East. Calculations are based on the average 1000 seed weight given in the Seed Information Database (SID) of the Royal Botanic Gardens Kew [67]. Numbers in brackets give the number of extant Near Eastern species or subspecies for which measurements were available. The average seed weight for the single species is also based on multiple measurements from different accessions; for information on these data please see SID.
(TIF)

S3 Fig PRISMA flow diagram showing the impact of the selection criteria on the screened datasets. (TIF)

S4 Fig The composition of Poaceae remains in the samples used in the correspondence analysis. Note that the strong temporal trend throughout the sequence is also visible in the Poaceae remains. Whereas the older samples from AH VII and VI have low percentages of small grains (right end of axis 1), samples from the middle part of the analyzed sequence are dominated by these taxa. Emmer wheat chaff remains characterize samples from AH III and II, where small grains are still very abundant (left end of axis 1). Large to medium seeded wild grasses are in all samples more abundant than grains of *H*. *spontaneum* and *Aegilops* sp. together.
(TIF)

S5 Fig Detailed key for the sites included in the temporal and spatial analysis of grain proportions. (TIF)

S6 Fig PRISMA checklist for the systematic review. (TIF)
