# Supplementary material for: A systematic review of wild grass exploitation in relation to emerging cereal cultivation throughout the Epipalaeolithic and aceramic Neolithic of the Fertile Crescent
Source: PLoS One. 2018 Jan 2;13(1):e0189811. doi: 10.1371/journal.pone.0189811 (PMC5749723; doi:10.1371/journal.pone.0189811)
Supplement: S1 Table — Based on the Seed Information Database of the Royal Botanic Gardens Kew [67]. (DOCX) [file pone.0189811.s001.docx]

| **S1 Table.** **Average 1000 seed weight of Poaceae species.** Based on the Seed Information Database of the Royal Botanic Gardens Kew [67]. | |
| --- | --- |
| Taxon | Average 1000 |
|  | seed weight (g) |
| ***Aegilops* spp. mean** | **36.55** |
| *Aegilops bicornis* | 9.20 |
| *Aegilops crassa* | 17.31 |
| *Aegilops cylindrica* | 26.60 |
| *Aegilops geniculata* | 71.50 |
| *Aegilops juvenalis* | 18.76 |
| *Aegilops kotschyi* | 47.60 |
| *Aegilops peregrina* | 87.80 |
| *Aegilops speltoides* | 22.85 |
| *Aegilops tauschii* | 27.30 |
| ***Aeluropus* spp. mean** | **0.23** |
| *Aeluropus lagopoides* | 0.26 |
| *Aeluropus littoralis* | 0.20 |
| ***Agrostis* spp. mean** | **0.09** |
| *Agrostis canina* | 0.06 |
| *Agrostis capillaris* | 0.08 |
| *Agrostis castellana* | 0.17 |
| *Agrostis gigantea* | 0.08 |
| *Agrostis stolonifera* | 0.07 |
| ***Alopecurus* spp. mean** | **1.02** |
| *Alopecurus aequalis* | 0.17 |
| *Alopecurus apiatus* | 1.50 |
| *Alopecurus arundinaceus* | 0.50 |
| *Alopecurus bulbosus* | 0.46 |
| *Alopecurus geniculatus* | 0.28 |
| *Alopecurus gerardii* | 1.17 |
| *Alopecurus glacialis* | 0.66 |
| *Alopecurus myosuroides* | 1.99 |
| *Alopecurus pratensis* | 0.90 |
| *Alopecurus rendlei* | 2.66 |
| *Alopecurus textilis* | 1.73 |
| *Alopecurus utriculatus* | 0.87 |
| *Alopecurus vaginatus* | 0.33 |
| ***Avena* spp. mean** | **26.12** |
| *Avena barbata* | 11.00 |
| *Avena fatua* | 28.00 |
| *Avena sterilis* | 50.00 |
| *Avena ventricosa* | 15.48 |
| ***Bromus* spp. mean** | **4.43** |
| *Bromus alopecurus* | 2.20 |
| *Bromus arvensis* | 2.20 |
| *Bromus danthoniae* | 5.67 |
| *Bromus fasciculatus* | 1.76 |
| *Bromus japonicus* | 2.61 |
| *Bromus lanceolatus* | 3.93 |
| *Bromus madritensis* | 3.33 |
| **S1 Table.** Continued. | |
| Taxon | Average 1000 |
|  | seed weight (g) |
| *Bromus pectinatus* | 4.10 |
| *Bromus rigidus* | 10.19 |
| *Bromus sterilis* | 9.50 |
| *Bromus tectorum* | 3.20 |
| ***Crypsis* spp. mean** | **0.27** |
| *Crypsis aculeata* | 0.29 |
| *Crypsis acuminata* | 0.19 |
| *Crypsis alupecuroides* | 0.20 |
| *Crypsis factorovskyi* | 0.33 |
| *Crypsis minuartioides* | 0.37 |
| *Crypsis schoenoides* | 0.24 |
| ***Echinaria capitata*** | **5.50** |
| ***Echinochloa* spp. mean** | **1.15** |
| *Echinochloa colonum* | 0.80 |
| *Echinochloa crus-galli* | 1.50 |
| ***Eragrostis* spp. mean** | **0.07** |
| *Eragrostis barrelieri* | 0.09 |
| *Eragrostis cilianensis* | 0.08 |
| *Eragrostis japonica* | 0.03 |
| *Eragrostis minor* | 0.09 |
| *Eragrostis pilosa* | 0.07 |
| ***Eremopyrum* spp. mean** | **3.09** |
| *Eremopyrum bonaepartis* | 3.95 |
| *Eremopyrum distans* | 4.98 |
| *Eremopyrum orientale* | 0.34 |
| ***Hordeum* spp. mean** | **13.33** |
| *Hordeum brevisubulatum* | 3.52 |
| *Hordeum bulbosum* | 11.00 |
| *Hordeum glaucum* | 3.24 |
| *Hordeum leporinum* | 6.84 |
| *Hordeum marinum* | 11.80 |
| *Hordeum murinum* | 23.40 |
| *Hordeum secalinum* | 6.43 |
| *Hordeum spontaneum* | 41.70 |
| *Hordeum violaceum* | 12.00 |
| ***Lolium* spp. mean** | **5.74** |
| *Lolium multiflorum* | 2.90 |
| *Lolium perenne* | 2.20 |
| *Lolium persicum* | 9.64 |
| *Lolium rigidum* | 3.45 |
| *Lolium temulentum* | 10.50 |
| ***Panicum* spp. mean** | **1.19** |
| *Panicum antidotale* | 1.00 |
| *Panicum maximum* | 1.10 |
| *Panicum repens* | 0.65 |
| *Panicum turgidum* | 2.00 |

| **S1 Table.** Continued. | |
| --- | --- |
| Taxon | Average 1000 |
|  | seed weight (g) |
| ***Phalaris* spp. mean** | **2.05** |
| *Phalaris aquatica* | 1.69 |
| *Phalaris arundinacea* | 0.70 |
| *Phalaris brachystachis* | 1.90 |
| *Phalaris canariensis* | 5.20 |
| *Phalaris minor* | 1.50 |
| *Phalaris paradoxa* | 1.30 |
| ***Phleum* spp. mean** | **0.28** |
| *Phleum alpinum* | 0.41 |
| *Phleum arenarium* | 0.20 |
| *Phleum bertolonii* | 0.41 |
| *Phleum exaratum* | 0.25 |
| *Phleum montanum* | 0.12 |
| *Phleum phleoides* | 0.15 |
| *Phleum pratense* | 0.40 |
| ***Poa* spp. mean** | **0.31** |
| *Poa alpina* | 0.36 |
| *Poa angustifolia* | 0.22 |
| *Poa annua* | 0.30 |
| *Poa bulbosa* | 0.90 |
| *Poa caucasica* | 0.25 |
| *Poa compressa* | 0.20 |
| *Poa nemoralis* | 0.20 |
| *Poa pratensis* | 0.25 |
| *Poa trivialis* | 0.10 |
| ***Puccinellia* spp. mean** | **0.17** |
| *Puccinellia bulbosa* | 0.10 |
| *Puccinellia ciliata* | 0.20 |
| *Puccinellia distans* | 0.20 |
|  |  |
| **S1 Table.** Continued. | |
| Taxon | Average 1000 |
|  | seed weight (g) |
| ***Secale* spp. mean** | **8.17** |
| *Secale anatolicum* | 3.70 |
| *Secale montanum* | 12.10 |
| *Secale sylvestre* | 8.70 |
| ***Setaria* spp. mean** | **1.82** |
| *Setaria glauca* | 3.75 |
| *Setaria verticillata* | 0.70 |
| *Setaria viridis* | 1.00 |
| ***Sporobolus* spp. mean** | **0.14** |
| *Sporobolus spicatus* | 0.14 |
| *Sporobolus virginicus* | 0.15 |
| ***Stipa* spp. mean** | **8.97** |
| *Stipa arabica* | 2.79 |
| *Stipa bromoides* | 5.15 |
| *Stipa capensis* | 1.68 |
| *Stipa capillata* | 8.20 |
| *Stipa caucasica* | 5.61 |
| *Stipa holoserica* | 8.65 |
| *Stipa lagascae* | 9.10 |
| *Stipa parviflora* | 1.19 |
| *Stipa pennata* | 20.46 |
| *Stipa pulcherrima* | 25.95 |
| *Stipa tirsa* | 14.65 |
| *Stipa turkestanica* | 4.15 |
| ***Taeniatherum* spp. mean** | **6.19** |
| *Taeniatherum caput-medusae* | 7.03 |
| *Taeniatherum crinitum* | 5.35 |
| ***Triticum* spp. mean** | **27.21** |
| *Triticum boeoticum* | 13.00 |
| *Triticum diccoccoides* | 41.41 |
