# Supplementary material for: A systematic review of wild grass exploitation in relation to emerging cereal cultivation throughout the Epipalaeolithic and aceramic Neolithic of the Fertile Crescent
Source: PLoS One. 2018 Jan 2;13(1):e0189811. doi: 10.1371/journal.pone.0189811 (PMC5749723; doi:10.1371/journal.pone.0189811)
Supplement: S2 Table — (DOCX) [file pone.0189811.s002.docx]

**S2 Table.** Analyzed flotation samples and identified plant remains from AH II-VII from the deep sounding of Chogha Golan.

| Archaeological Horizon (AH) | II | | III | | IV | | V | | VI | | VII | |
| --- | --- | --- | --- | --- | --- | --- | --- | --- | --- | --- | --- | --- |
| Floated sediments (l) | 178 | | 58 | | 32 | | 56 | | 40 | | 54 | |
| Samples | 15 | | 5 | | 3 | | 5 | | 5 | | 5 | |
| Mean sample volume (l) | 12 | | 12 | | 11 | | 11 | | 8 | | 11 | |
| Items per liter soil | 17.5 | | 73.2 | | 300.9 | | 54.7 | | 119.1 | | 33.8 | |
| Number of identified taxa | 58 | | 68 | | 66 | | 53 | | 64 | | 67 | |
| Total number of analyzed plant remains | 3107 | | 4246 | | 9628 | | 3061 | | 4765 | | 1824 | |
|  | *n* | % | *n* | % | *n* | % | *n* | % | *n* | % | *n* | % |
| **Amaranthaceae** |  |  |  |  |  |  |  |  |  |  |  |  |
| *Atriplex* sp. | 1 | 0.03 | - | - | 3 | 0.03 | 3 | 0.10 | 255 | 5.35 | 32 | 1.75 |
| cf. *Atriplex* sp. | - | - | - | - | - | - | 3 | 0.10 | - | - | 3 | 0.16 |
| *Atriplex* sp. bracts | - | - | - | - | - | - | - | - | 35 | 0.73 | 4 | 0.22 |
| *Chenopodium* sp. | - | - | - | - | 1 | 0.01 | - | - | - | - | - | - |
| cf. *Salsola* sp. | - | - | - | - | - | - | - | - | - | - | 2 | 0.11 |
| *Suaeda* sp. | 1 | 0.03 | - | - | 8 | 0.08 | - | - | 16 | 0.34 | 10 | 0.55 |
| Amaranthaceae indet. | 4 | 0.13 | 4 | 0.09 | 9 | 0.09 | 12 | 0.39 | 1 | 0.02 | 8 | 0.44 |
| **Anacardiaceae** |  |  |  |  |  |  |  |  |  |  |  |  |
| *Pistacia* sp. | 38 | 1.22 | 70 | 1.65 | 38 | 0.39 | 79 | 2.58 | 10 | 0.21 | 37 | 2.03 |
| **Apiaceae** |  |  |  |  |  |  |  |  |  |  |  |  |
| Apiaceae type 1 | - | - | 1 | 0.02 | - | - | 1 | 0.03 | 14 | 0.29 | 6 | 0.33 |
| *Coriandrum* sp. | - | - | 1 | 0.02 | - | - | - | - | - | - | - | - |
| Apiaceae indet. | - | - | 1 | 0.02 | - | - | - | - | - | - | - | - |
| **Asparagaceae** |  |  |  |  |  |  |  |  |  |  |  |  |
| *Bellevalia*/*Muscari*/*Ornithogalum* | 11 | 0.35 | 6 | 0.14 | 17 | 0.18 | 26 | 0.85 | 117 | 2.46 | 25 | 1.37 |
| *Muscari*/*Ornithogalum* | - | - | 1 | 0.02 | - | - | - | - | - | - | 1 | 0.05 |
| **Asteraceae** | - | - | - | - | - | - | - | - | - | - | - | - |
| *Centaurea* sp. | 2 | 0.06 | 7 | 0.16 | 20 | 0.21 | 9 | 0.29 | 60 | 1.26 | 6 | 0.33 |
| cf. *Centaurea* sp. | - | - | 1 | 0.02 | 2 | 0.02 | - | - | - | - | 2 | 0.11 |
| *Cirsium* type | - | - | - | - | 2 | 0.02 | - | - | - | - | 1 | 0.05 |
| Asteraceae indet. | 3 | 0.10 | 2 | 0.05 | 1 | 0.01 | - | - | - | - | 4 | 0.22 |
| **Boraginaceae** |  |  |  |  |  |  |  |  |  |  |  |  |
| *Buglossoides arvensis* (uncharred) | 7 | 0.23 | 1 | 0.02 | 1 | 0.01 | - | - | 8 | 0.17 | - | - |
| *Buglossoides tenuiflora* (uncharred) | 5 | 0.16 | 1 | 0.02 | - | - | - | - | - | - | 1 | 0.05 |
| *Heliotropium* sp. | 6 | 0.19 | 1 | 0.02 | 8 | 0.08 | 2 | 0.07 | 3 | 0.06 | 6 | 0.33 |
| **Brassicaceae** |  |  |  |  |  |  |  |  |  |  |  |  |
| *Alyssum* sp. | - | - | 1 | 0.02 | - | - | - | - | 126 | 2.64 | 14 | 0.77 |
| *Capsella*/*Descurainia* | 7 | 0.23 | - | - | 19 | 0.20 | 4 | 0.13 | 40 | 0.84 | 24 | 1.32 |
| *Erysimum*/*Sisymbrium* type | 2 | 0.06 | - | - | - | - | - | - | - | - | - | - |
| *Lepidium* sp. | - | - | 4 | 0.09 | 20 | 0.21 | 8 | 0.26 | 43 | 0.90 | 7 | 0.38 |
| cf. *Lepidium* sp. | - | - | 1 | 0.02 | - | - | 1 | 0.03 | - | - | - | - |
| Brassicaceae indet. | 3 | 0.10 | 20 | 0.47 | 1 | 0.01 | 2 | 0.07 | 4 | 0.08 | 6 | 0.33 |
| cf. Brassicaceae indet. | - | - | - | - | - | - | - | - | - | - | 4 | 0.22 |
| Brassicaceae silique fragments | - | - | 5 | 0.12 | 1 | 0.01 | - | - | 1 | 0.02 | 1 | 0.05 |
| **Caryophyllaceae** |  |  |  |  |  |  |  |  |  |  |  |  |
| *Dianthus sp.* | 1 | 0.03 | - | - | - | - | - | - | - | - | - | - |
| *Gypsophila* sp. | 2 | 0.06 | 2 | 0.05 | 26 | 0.27 | 7 | 0.23 | 39 | 0.82 | 6 | 0.33 |
| *Gypsophila*/*Silene* | 4 | 0.13 | 6 | 0.14 | 31 | 0.32 | 9 | 0.29 | 44 | 0.92 | 8 | 0.44 |
| *Silene* sp. | 1 | 0.03 | 1 | 0.02 | 21 | 0.22 | 7 | 0.23 | 8 | 0.17 | 5 | 0.27 |
| Caryophyllaceae indet. | 2 | 0.06 | 2 | 0.05 | 1 | 0.01 | - | - | - | - | - | - |
| **Cyperaceae** |  |  |  |  |  |  |  |  |  |  |  |  |
| *Bolboschoenus glaucus* | 34 | 1.09 | 20 | 0.47 | 35 | 0.36 | 17 | 0.56 | 18 | 0.38 | 12 | 0.66 |
| Cyperaceae indet. | 9 | 0.29 | 3 | 0.07 | 3 | 0.03 | 1 | 0.03 | 8 | 0.17 | 6 | 0.33 |
| **Euphorbiaceae** |  |  |  |  |  |  |  |  |  |  |  |  |
| *Euphorbia* type | 1 | 0.03 | 1 | 0.02 | - | - | - | - | - | - | - | - |
| **Fabaceae** |  |  |  |  |  |  |  |  |  |  |  |  |
| *Astragalus* sp. | 122 | 3.93 | 112 | 2.64 | 357 | 3.71 | 118 | 3.85 | 402 | 8.44 | 114 | 6.25 |
| *Lathyrus* type | 6 | 0.19 | 8 | 0.19 | 9 | 0.09 | 7 | 0.23 | 5 | 0.10 | 3 | 0.16 |
| *Lathyrus*/*Vicia* | 3 | 0.10 | 11 | 0.26 | 19 | 0.20 | 8 | 0.26 | 8 | 0.17 | 3 | 0.16 |
| *Lathyrus*/*Pisum*/*Vicia* | 40 | 1.29 | 43 | 1.01 | 150 | 1.56 | 76 | 2.48 | 233 | 4.89 | 45 | 2.47 |
| *Lens* sp. | 37 | 1.19 | 34 | 0.80 | 170 | 1.77 | 81 | 2.65 | 517 | 10.85 | 87 | 4.77 |
| *Medicago* sp. | 4 | 0.13 | 8 | 0.19 | 23 | 0.24 | 8 | 0.26 | 66 | 1.39 | 13 | 0.71 |
| cf. *Medicago* sp. | - | - | 1 | 0.02 | - | - | 1 | 0.03 | - | - | 3 | 0.16 |
| *Medicago radiata* | 23 | 0.74 | 17 | 0.40 | 43 | 0.45 | 21 | 0.69 | 18 | 0.38 | 9 | 0.49 |
| *Onobrychis* sp. | 1 | 0.03 | - | - | - | - | - | - | - | - | - | - |
| *Pisum* sp. | 1 | 0.03 | 3 | 0.07 | 23 | 0.24 | 7 | 0.23 | 7 | 0.15 | 1 | 0.05 |
| cf. *Pisum* sp. | - | - | 1 | 0.02 | - | - | - | - | 1 | 0.02 | 2 | 0.11 |
| *Trigonella astroites* type | 113 | 3.64 | 75 | 1.77 | 167 | 1.73 | 51 | 1.67 | 145 | 3.04 | 33 | 1.81 |
| *Trigonella* type 2 | 83 | 2.67 | 115 | 2.71 | 66 | 0.69 | 30 | 0.98 | 46 | 0.97 | 64 | 3.51 |
| *Trigonella foenum-graecum* type | - | - | 1 | 0.02 | 1 | 0.01 | - | - | 1 | 0.02 | - | - |
| *Trigonella*/*Astragalus* | 284 | 9.14 | 191 | 4.50 | 637 | 6.62 | 174 | 5.68 | 467 | 9.80 | 177 | 9.70 |
| *Vicia ervilia* | - | - | - | - | 1 | 0.01 | - | - | - | - | - | - |
| Fabaceae indet. large | 1 | 0.03 | 11 | 0.26 | 23 | 0.24 | 23 | 0.75 | 16 | 0.34 | 8 | 0.44 |
| Fabaceae indet. medium-small | 105 | 3.38 | 87 | 2.05 | 97 | 1.01 | 27 | 0.88 | 70 | 1.47 | 34 | 1.86 |
| **Lamiaceae** |  |  |  |  |  |  |  |  |  |  |  |  |
| cf. *Salvia* | - | - | - | - | 1 | 0.01 | - | - | - | - | - | - |
| *Ziziphora* sp. | - | - | 1 | 0.02 | 1 | 0.01 | - | - | - | - | - | - |
| Lamiaceae indet. | - | - | - | - | - | - | - | - | 4 | 0.08 | - | - |
| **Linaceae** |  |  |  |  |  |  |  |  |  |  |  |  |
| *Linum* sp. | - | - | - | - | 1 | 0.01 | - | - | - | - | - | - |
| **Malvaceae** |  |  |  |  |  |  |  |  |  |  |  |  |
| *Malva* sp. | 42 | 1.35 | 47 | 1.11 | 52 | 0.54 | 43 | 1.40 | 31 | 0.65 | 35 | 1.92 |
| **Papaveraceae** |  |  |  |  |  |  |  |  |  |  |  |  |
| *Fumaria* sp. | - | - | - | - | - | - | - | - | 1 | 0.02 | - | - |
| **Plantaginaceae** |  |  |  |  |  |  |  |  |  |  |  |  |
| *Plantago* sp. | - | - | - | - | - | - | - | - | - | - | 1 | 0.05 |
| **Poaceae** |  |  |  |  |  |  |  |  |  |  |  |  |
| *Aegilops* sp. | 20 | 0.64 | 24 | 0.57 | 28 | 0.29 | 19 | 0.62 | 93 | 1.95 | 20 | 1.10 |
| *Aegilops* sp. spikelets | 121 | 3.89 | 187 | 4.40 | 71 | 0.74 | 139 | 4.52 | 129 | 2.70 | 107 | 5.87 |
| *Avena* sp. | 13 | 0.42 | 54 | 1.27 | 259 | 2.69 | 80 | 2.61 | 117 | 2.46 | 60 | 3.29 |
| cf. *Avena* sp. | - | - | 12 | 0.28 | 42 | 0.44 | 9 | 0.29 | 15 | 0.31 | 16 | 0.88 |
| *Avena* sp. awn fragments | p |  | p |  | p |  | p |  | p |  | p |  |
| *Bromus* sp. | 14 | 0.45 | 29 | 0.68 | 123 | 1.28 | 34 | 1.11 | 79 | 1.66 | 34 | 1.86 |
| *Eremopyrum* sp. | - | - | - | - | - | - | - | - | 8 | 0.17 | 3 | 0.16 |
| *Eremopyrum*/*Agropyron* | 1 | 0.03 | 2 | 0.05 | 11 | 0.11 | 2 | 0.07 | - | - | 1 | 0.05 |
| *Hordeum* sp. | 23 | 0.74 | 24 | 0.57 | 46 | 0.48 | 59 | 1.93 | 181 | 3.80 | 49 | 2.69 |
| *Hordeum spontaneum* | 51 | 1.64 | 79 | 1.86 | 293 | 3.04 | 114 | 3.72 | 275 | 5.77 | 98 | 5.37 |
| *Hordeum* cf. *spontaneum* | 3 | 0.10 | 18 | 0.42 | 18 | 0.19 | 6 | 0.20 | 22 | 0.46 | 20 | 1.10 |
| *Hordeum spontaneum* spikelets | 111 | 3.57 | 166 | 3.91 | 372 | 3.86 | 116 | 3.79 | 112 | 2.35 | 112 | 6.14 |
| *Pennisetum* type | 3 | 0.10 | - | - | - | - | - | - | - | - | - | - |
| *Phalaris* sp. | 337 | 10.85 | 151 | 3.56 | 498 | 5.17 | 128 | 4.18 | 37 | 0.78 | 17 | 0.93 |
| *Phleum* type | 295 | 9.49 | 977 | 23.01 | 2943 | 30.57 | 776 | 25.35 | 109 | 2.29 | 30 | 1.64 |
| *Poa bulbosa* bulbils | - | - | 1 | 0.02 | 1 | 0.01 | 1 | 0.03 | 76 | 1.59 | 6 | 0.33 |
| *Taeniatherum caput-medusae* | 3 | 0.10 | 14 | 0.33 | 26 | 0.27 | 14 | 0.46 | 10 | 0.21 | 3 | 0.16 |
| *Taeniatherum caput-medusae* spikelets | 5 | 0.16 | 12 | 0.28 | 10 | 0.10 | 9 | 0.29 | 1 | 0.02 | 2 | 0.11 |
| Triticoid type | 17 | 0.55 | 41 | 0.97 | 113 | 1.17 | 56 | 1.83 | 49 | 1.03 | 11 | 0.60 |
| cf. *Triticum* sp. | 6 | 0.19 | - | - | - | - | - | - | - | - | - | - |
| *Triticum* *boeoticum* spikelets | 11 | 0.35 | 7 | 0.16 | - | - | - | - | - | - | 1 | 0.05 |
| *Triticum* *dicoccoides*/*dicoccum* spikelets | 77 | 2.45 | 16 | 0.38 | 2 | 0.02 | - | - | 1 | 0.02 | 5 | 0.27 |
| *Triticum* sp. spikelets | 165 | 5.34 | 25 | 0.59 | 3 | 0.03 | - | - | 3 | 0.06 | 14 | 0.77 |
| Indeterminate spikelet type | 60 | 1.93 | 80 | 1.88 | 48 | 0.50 | 129 | 4.21 | 26 | 0.55 | 57 | 3.13 |
| Poaceae indet. glumes | 47 | 1.51 | 8 | 0.19 | - | - | 3 | 0.10 | - | - | - | - |
| Poaceae indet. spikelets | 3 | 0.10 | 8 | 0.19 | 4 | 0.04 | 13 | 0.42 | 8 | 0.17 | 19 | 1.04 |
| Poaceae indet. large | 66 | 2.12 | 33 | 0.78 | 59 | 0.61 | 12 | 0.39 | 21 | 0.44 | 10 | 0.55 |
| Poaceae indet. medium | 163 | 5.25 | 129 | 3.04 | 214 | 2.22 | 51 | 1.67 | 69 | 1.45 | 60 | 3.29 |
| Poaceae indet. small | 337 | 10.85 | 974 | 22.94 | 2054 | 21.33 | 322 | 10.52 | 198 | 4.16 | 100 | 5.48 |
| **Polygonaceae** |  |  |  |  |  |  |  |  |  |  |  |  |
| *Rumex*/*Polygonum* | 2 | 0.06 | - | - | - | - | - | - | - | - | - | - |
| **Ranunculaceae** |  |  |  |  |  |  |  |  |  |  |  |  |
| *Adonis* sp. | - | - | 2 | 0.05 | 2 | 0.02 | - | - | 4 | 0.08 | - | - |
| **Rubiaceae** | - | - | - | - | - | - | - | - | - | - | - | - |
| *Galium* sp. | 5 | 0.16 | 3 | 0.07 | 3 | 0.03 | 9 | 0.29 | 8 | 0.17 | 1 | 0.05 |
| **Indeterminate types** |  |  |  |  |  |  |  |  |  |  |  |  |
| Indeterminate type 1 | - | - | 2 | 0.05 | - | - | - | - | - | - | - | - |
| Indeterminate type 2 | - | - | 3 | 0.07 | - | - | - | - | 1 | 0.02 | - | - |
| Indeterminate type 3 | - | - | - | - | 4 | 0.04 | - | - | 4 | 0.08 | - | - |
| Indeterminate type 4 | - | - | - | - | 4 | 0.04 | - | - | - | - | - | - |
| Indeterminate type 5 | - | - | - | - | - | - | - | - | 3 | 0.06 | - | - |
| Indeterminate type 6 | - | - | - | - | 8 | 0.08 | - | - | 25 | 0.52 | 8 | 0.44 |
| Indeterminate | 139 | 4.47 | 236 | 5.56 | 260 | 2.70 | 94 | 3.07 | 193 | 4.05 | 87 | 4.77 |

If no other information is given, identifications represent charred fruits or seeds. Note that the data from AH II were already published elsewhere [44]; p = present.
