# Supplementary material for: A systematic review of wild grass exploitation in relation to emerging cereal cultivation throughout the Epipalaeolithic and aceramic Neolithic of the Fertile Crescent
Source: PLoS One. 2018 Jan 2;13(1):e0189811. doi: 10.1371/journal.pone.0189811 (PMC5749723; doi:10.1371/journal.pone.0189811)
Supplement: S3 Table — (DOCX) [file pone.0189811.s003.docx]

**S3 Table.** Coding of the variables in the correspondence analysis.

| Number/Code | Taxon/category |
| --- | --- |
| 1 | *Aegilops* grain |
| 2 | *Aegilops* spikelets |
| 3 | Amaranthaceae |
| 4 | Asteraceae |
| 5 | *Astragalus*/*Medicago*/*Trigonella* |
| 6 | *Avena* |
| 7 | *Bolboschoenus glaucus* |
| 8 | Brassicaceae |
| 9 | *Bromus* |
| 10 | Cyperaceae |
| 11 | Fabaceae inet. large |
| 12 | Fabaceae indet. medium-small |
| 13 | *Galium* |
| 14 | *Heliotropium* |
| 15 | *Hordeum* |
| 16 | *Hordeum spontaneum* grain |
| 17 | *Hordeum spontaneum* spikelets |
| 18 | Indet. spikelet type |
| 19 | *Lathyrus*/*Pisum*/*Vicia* |
| 20 | *Lens* |
| 21 | *Malva* |
| 22 | *Phalaris* |
| 23 | *Phleum* type |
| 24 | *Pistacia* |
| 25 | *Pisum* |
| 26 | Poaceae chaff indet. |
| 27 | Poaceae indet. large |
| 28 | Poaceae indet. medium |
| 29 | Poaceae indet. small |
| 30 | *Silene*/*Gypsophila* |
| 31 | *Taeniatherum caput-medusae* grain |
| 32 | *Taeniatherum caput-medusae* spikelets |
| 33 | Triticoid type |
| Agr/Erem | *Agropyron*/*Eremopyrum* |
| Bell/Mus/Orni | *Bellevalia*/*Muscari*/*Ornithogalum* |
| BugArv | *Buglossoides arvensis* |
| BugTen | *Buglossoides tenuiflora* |
| Cap/Des | *Capsella*/*Descurainia* |
| Caryop | Caryophyllaceae |
| PoaBulb | *Poa bulbosa* bulbils |
| TritSpik | *Triticum* spikelets |
