# Supplementary figures and images for: A systematic review of wild grass exploitation in relation to emerging cereal cultivation throughout the Epipalaeolithic and aceramic Neolithic of the Fertile Crescent
Source: PLoS One. 2018 Jan 2;13(1):e0189811. doi: 10.1371/journal.pone.0189811 (PMC5749723; doi:10.1371/journal.pone.0189811)

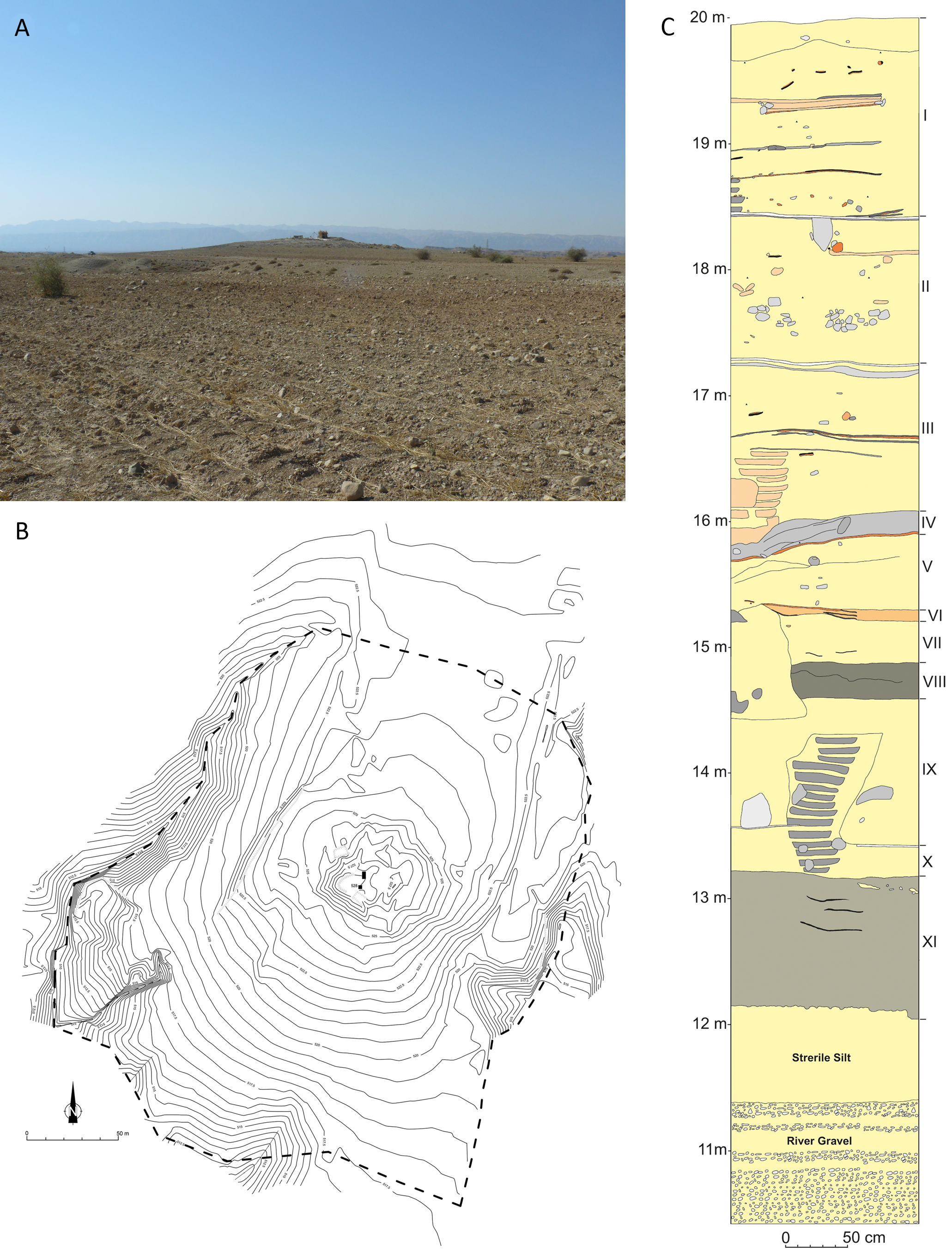

Supplement: S1 Fig — (A) The landscape around Chogha Golan; (B) outline of the site with the location of the deep sounding and excavation Area A in the center of the tell; (C) the south profile of the deep sounding showing Archaeological Horizons I to XI and the related z-values. Figures and photos by M. Zeidi. (TIF) [file pone.0189811.s004.tif]

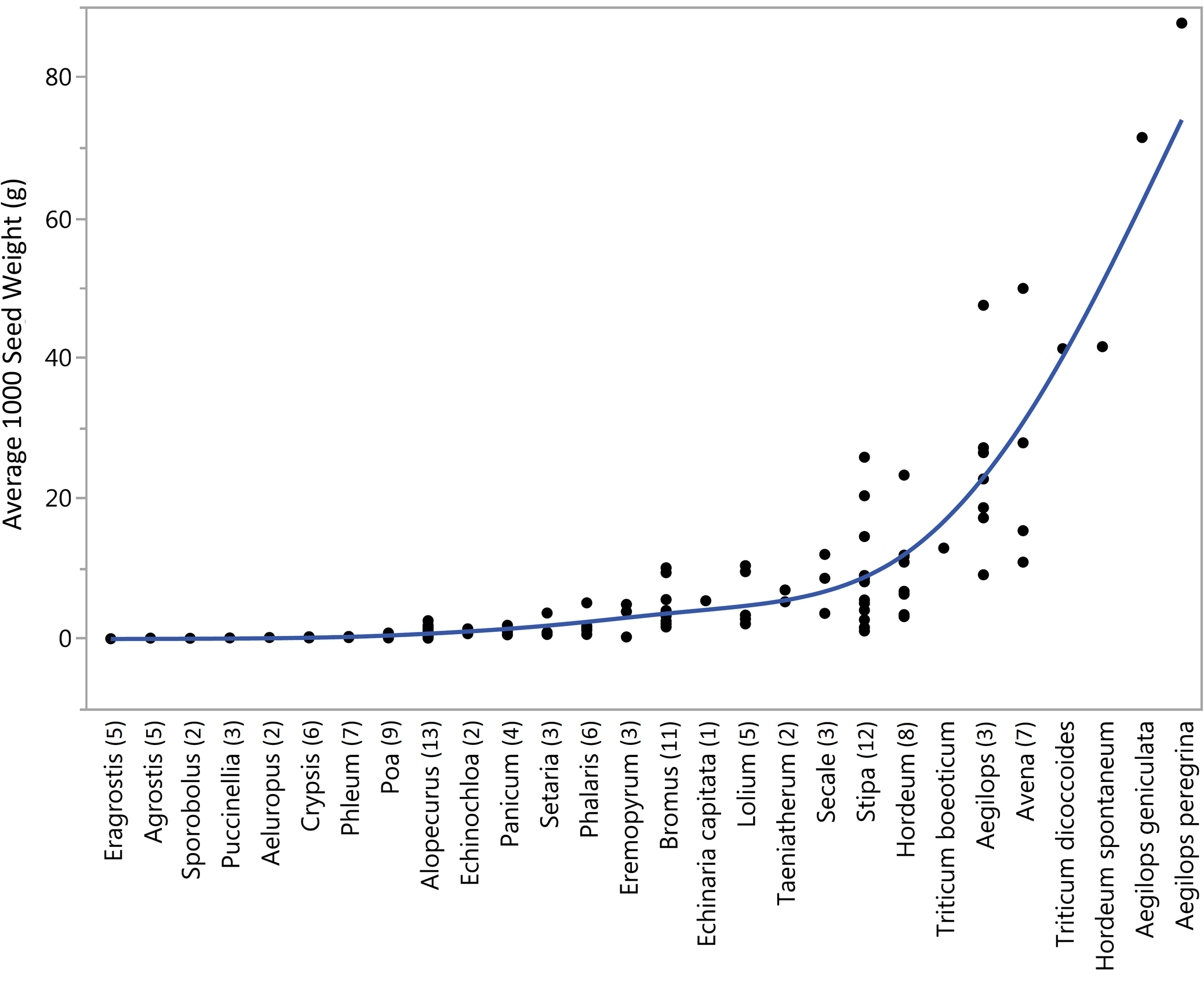

Supplement: S2 Fig — Calculations are based on the average 1000 seed weight given in the Seed Information Database (SID) of the Royal Botanic Gardens Kew [67]. Numbers in brackets give the number of extant Near Eastern species or subspecies for which measurements were available. The average seed weight for the single species is also based on multiple measurements from different accessions; for information on these data please see SID. (TIF) [file pone.0189811.s005.tif]

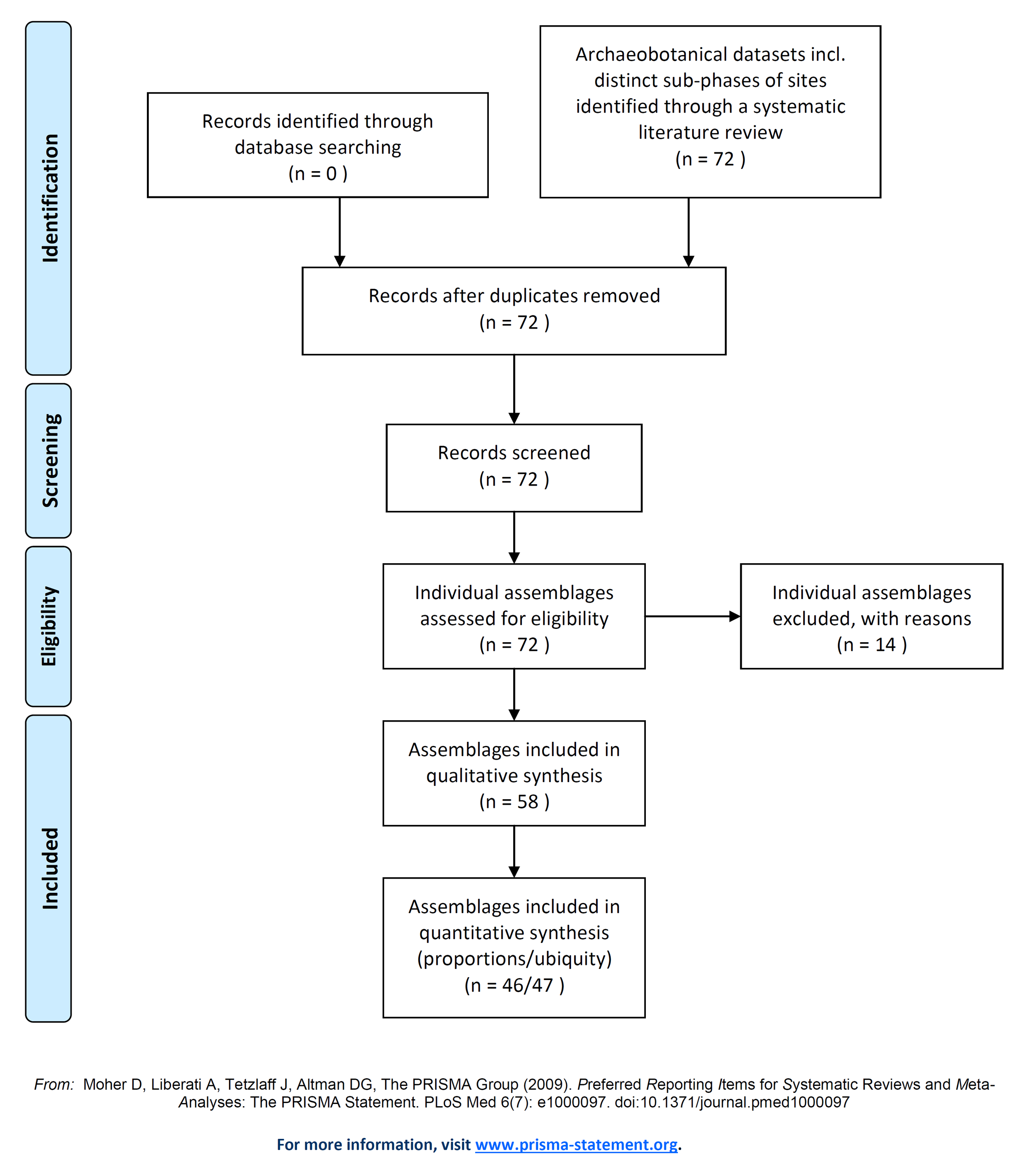

Supplement: S3 Fig — (TIF) [file pone.0189811.s006.tif]

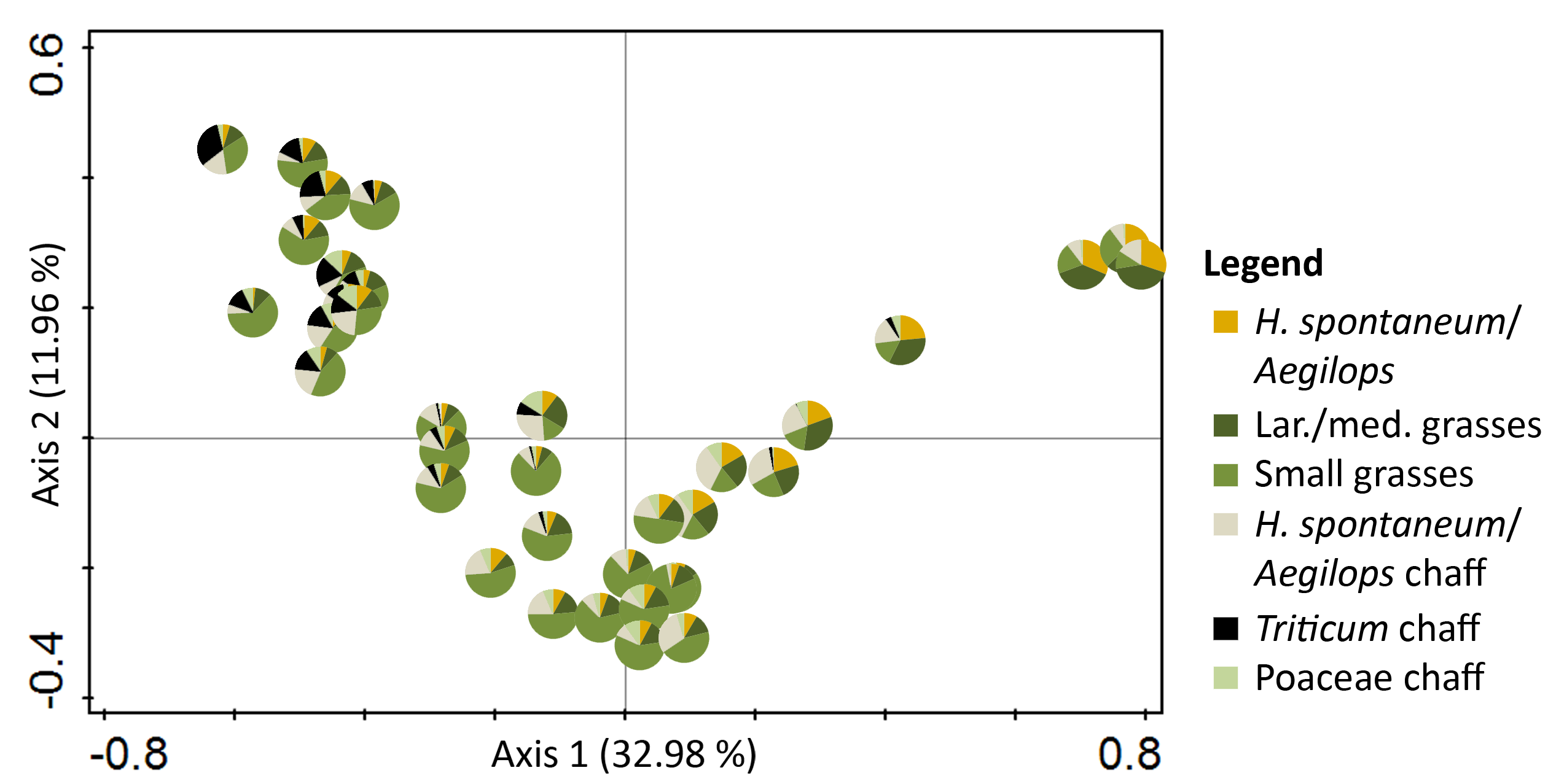

Supplement: S4 Fig — Note that the strong temporal trend throughout the sequence is also visible in the Poaceae remains. Whereas the older samples from AH VII and VI have low percentages of small grains (right end of axis 1), samples from the middle part of the analyzed sequence are dominated by these taxa. Emmer wheat chaff remains characterize samples from AH III and II, where small grains are still very abundant (left end of axis 1). Large to medium seeded wild grasses are in all samples more abundant than grains of H. spontaneum and Aegilops sp. together. (TIF) [file pone.0189811.s007.tif]

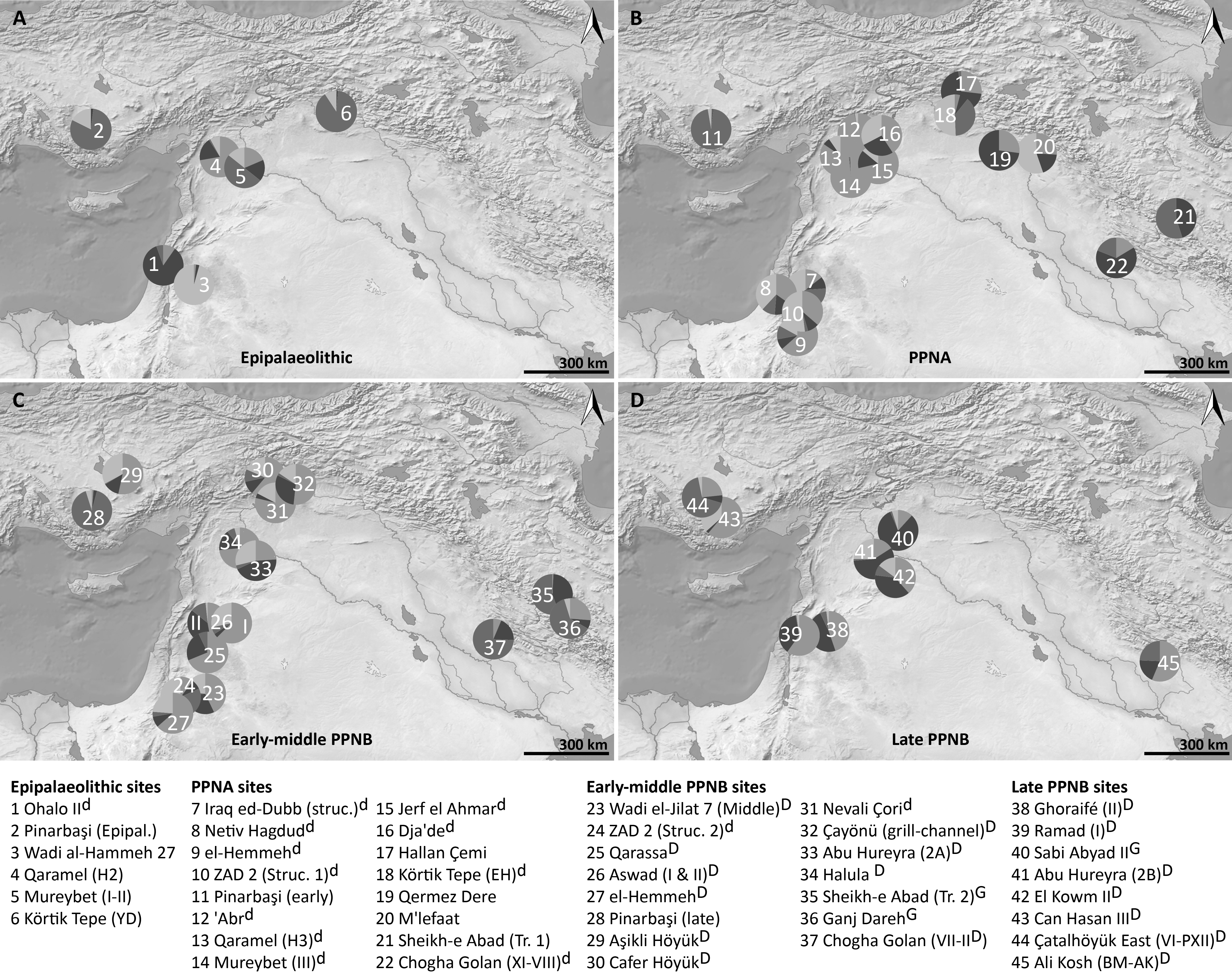

Supplement: S5 Fig — (TIF) [file pone.0189811.s008.tif]

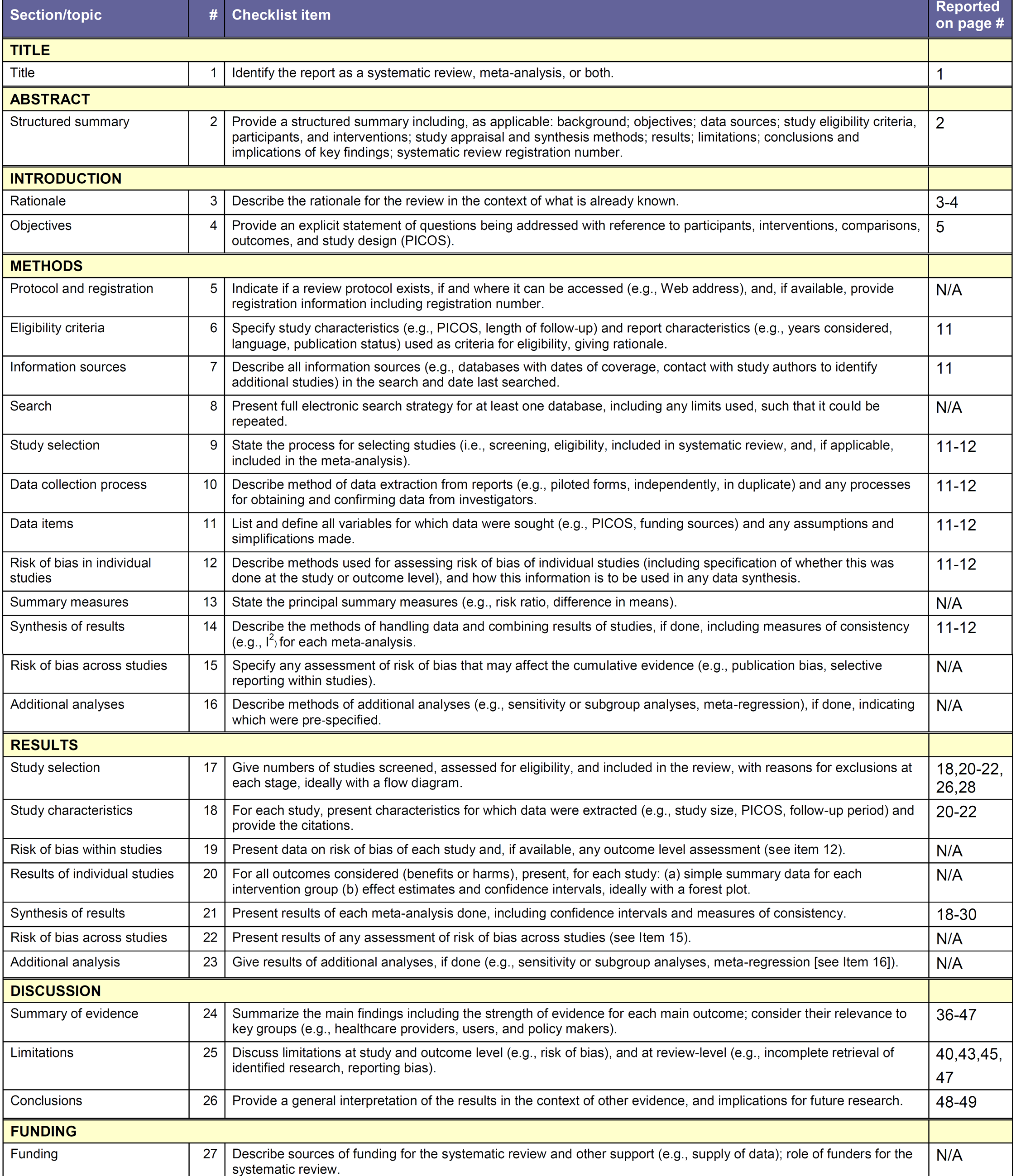

Supplement: S6 Fig — (TIF) [file pone.0189811.s009.tif]
